# Highly Hydrophobic Films of Engineered Silk Proteins by a Simple Deposition Method

**DOI:** 10.1021/acs.langmuir.2c03442

**Published:** 2023-03-16

**Authors:** Teemu Välisalmi, Nelmary Roas-Escalona, Kristoffer Meinander, Pezhman Mohammadi, Markus B. Linder

**Affiliations:** † Department of Bioproducts and Biosystems, School of Chemical Engineering, Aalto University, FI-00076 Aalto, Finland; ‡ VTT Technical Research Centre of Finland, Limited (VTT), FI-02044 Espoo, Finland; § Centre of Excellence in Life-Inspired Hybrid Materials (LIBER), Aalto University, Post Office Box 16100, 00076 Aalto, Finland

## Abstract

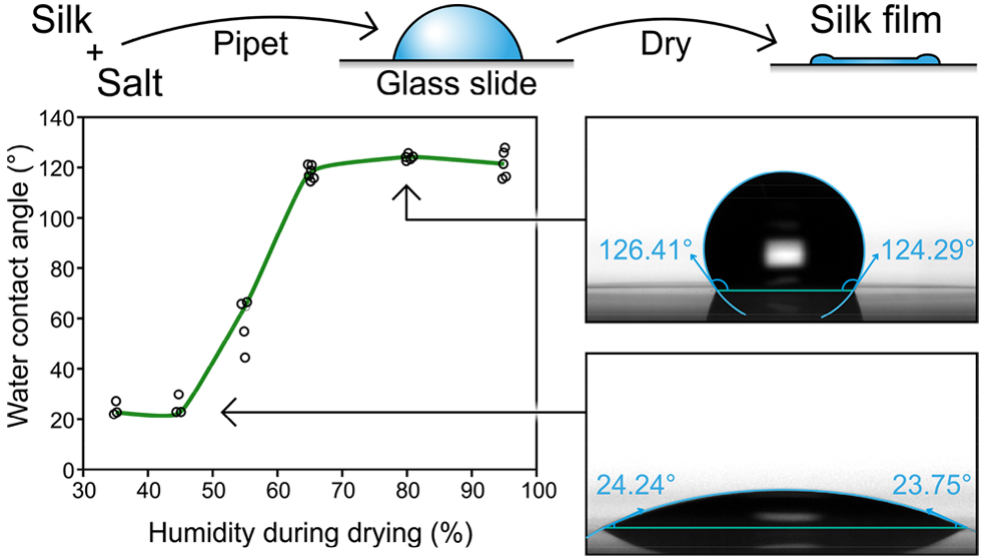

Molecular engineering of protein structures offers a uniquely versatile route for novel functionalities in materials. Here, we describe a method to form highly hydrophobic thin films using genetically engineered spider silk proteins. We used structurally engineered protein variants containing ADF3 and AQ12 spider silk sequences. Wetting properties were studied using static and dynamic contact angle measurements. Solution conditions and the surrounding humidity during film preparation were key parameters to obtain high hydrophobicity, as shown by contact angles in excess of 120°. Although the surface layer was highly hydrophobic, its structure was disrupted by the added water droplets. Crystal-like structures were found at the spots where water droplets had been placed. To understand the mechanism of film formation, different variants of the proteins, the topography of the films, and secondary structures of the protein components were studied. The high contact angle in the films demonstrates that the conformations that silk proteins take in the protein layer very efficiently expose their hydrophobic segments. This work reveals a highly amphiphilic nature of silk proteins and contributes to an understanding of their assembly mechanisms. It will also help in designing diverse technical uses for recombinant silk.

## 1 Introduction

Engineered recombinant proteins show a large potential as high-performance chemicals that will be needed for a societal transition toward sustainable materials with decreased environmental impact. A great challenge is to understand how to structurally design proteins to adapt them for specific functions. Amphiphilicity and hydrophobicity are examples of such functions. These are needed in, for example, wetting resistant coatings, stabilization of emulsions, and formulations for drug delivery.^1−6^ Because protein materials can be produced using microbes requiring only simple growth media and can be processed in aqueous conditions, they have the potential to allow savings in manufacturing costs and decreased environmental burden compared to current alternatives.^3,7−9^ Spider silk proteins have emerged as one of the most promising targets for research with the recent development of high-yield recombinant expression systems and impressive properties as fiber materials.^9^ They have also been successfully used in composite materials as the adhesive matrix.^10,11^ For further developments of new spider-silk-based materials, a deeper understanding of their molecular assembly mechanisms will be needed.

The mechanical properties of spider silk proteins originate from a unique mix of crystalline and amorphous structures.^5,12,13^ They have a middle section that consists of repeats of alanine-rich regions that are prone to crystallize and glycine-rich repeats that are more amorphous.^5,12,13^ The middle section of the native spider silk protein is flanked by N- and C-terminal domains that are thought to affect solubility and control dimerization of the silk proteins.^9,13^ Dependent upon conditions, the secondary structure of the silk protein can switch from an initially disordered to a tightly packed β-sheet-rich structure.^12−14^ Conditions that induce this change *in vivo* are pH, solutes like salt (e.g., potassium phosphate), and mechanical shear.^12,14,15^ In addition, *in vitro* conditions that have also been used to generate this structural shift include the temperature and solvents, like methanol.^12,14,15^ Importantly, spider silk proteins can undergo liquid–liquid phase separation (LLPS), which is thought to affect the molecular structuring during assembly into functional materials.^16,17^ During LLPS, the silk protein solution spontaneously separates into two phases: one that is highly concentrated and one that is dilute in protein. This phenomenon is thought to be driven by weak macromolecular interactions. Similar to the change into the β-sheet-rich secondary structure, LLPS is also influenced by various conditions, such as pH, silk protein concentration, and additives, like salt.^16−18^

One strategy for structural engineering of spider silk proteins is to make a triblock structure similar to the native spider silk protein with a repetitive mid-block flanked by two terminal domains.^9,15,16,19^ In such a strategy, the mid-block is a repetitive sequence of spider silk protein, such as ADF3^16,20^ from *Araneus diadematus* or the engineered sequence AQ12 that contains 12 consensus repeats derived from the native ADF3 sequence.^21^ The terminal domains can be native spider silk terminal domains, but it has been shown that replacing them with other globular domains provides a way to modify the functionality of the proteins,^16,18,22^ thereby opening more possibilities for engineering the silk protein construct.

Native and engineered silk proteins have previously been used to produce a variety of different coatings. Native silk proteins produced by *Bombyx mori*, i.e., fibroins, have been used to cast films that were then modified to, for example, enhance cell adhesion for tissue engineering,^23,24^ induce the transition into the β-sheet structure to increase film strength,^25^ or to add conductivity for use in sensors.^26^ Using protein engineering, spider silk proteins have been modified with peptides for antimicrobial coatings^27^ or for coatings with enhanced cell adhesion by adding recognition sites.^19^ The stability of coatings in aqueous environments has been improved using various post-treatments.^14,15^ Studies reporting the wetting properties of fibroin and engineered spider silk films have generally found them to be hydrophilic.^5,19,23,24,28,29^ In one study, the effects of different solvents as post-treatments of engineered spider silk based on the ADF4 protein were evaluated. It was found that hydrophobic silk films with an average water contact angle (CA) of 113° could be made by first preparing the film on a glass support in aqueous solution and then post-treating the film with methanol. It was proposed that the post-treatment rendered the film hydrophobic as a result of the formation of hydrophobic β-sheet crystals on the surface.^5^ In another study, they prepared porous fibroin films by introducing polystyrene beads in the film during casting and, afterward, dissolving the beads with toluene, which resulted in hydrophobic films (average CA of ∼120°) with a rough surface topography.^28^ In both cases, the extensive physical and chemical treatments led to hydrophobicity but with different film properties.

The water repellence of hydrophobic surfaces originates from two factors: the intrinsic hydrophobicity of nonpolar surface-exposed groups and the surface topography.^30−32^ A flat and smooth surface with nonpolar chemistries, such as methyl (−CH_3_) or fluoromethyl (−CF_3_), is expected to have CAs as a result of intrinsic hydrophobicity of maximally about 120°.^30,33−38^ With a suitable surface topography, such surfaces become more hydrophobic, even reaching superhydrophobicity (CA of >150°).^36−39^ There are two models that describe the wetting of structured surfaces: the Cassie–Baxter and the Wenzel models.^30,34,37,38^ In the Cassie–Baxter model, the liquid droplet lays on top of the surface topographic structure, with air filling the gaps in between, while in the Wenzel model, the liquid is in contact with all of the surface under the droplet.^30,34,37,38^ Cassie–Baxter surfaces can be described as “non-sticky” because droplets easily roll off the surface.^37,38^ In contrast, Wenzel surfaces are “sticky” because, even at high tilting angles, droplets can cling to the surface.^38^ If a surface material lacks intrinsic hydrophobicity, a higher surface roughness decreases the water CA, leading to high wettability.^40^

In this study, we describe a simple method for the casting of hydrophobic silk films from an aqueous solution without the need of protein denaturation or use of post-treatments. Different structural variants of ADF3 and AQ12 were used. Hydrophobicity was quantified by the contact angle between a drop of water and the silk films and was measured with an optical tensiometer. We demonstrate how salts can be used to increase hydrophobicity and how the relative humidity plays a role in the casting process. The films were studied with scanning electron microscopy (SEM), atomic force microscopy (AFM), circular dichroism (CD), and X-ray photoelectron spectroscopy (XPS). We show how the film behaves in dynamic contact angle measurement and how water affects the film structure.

## 2 Materials and Methods

### 2.1 Silk-Like Protein Constructs

**Figure 1:**
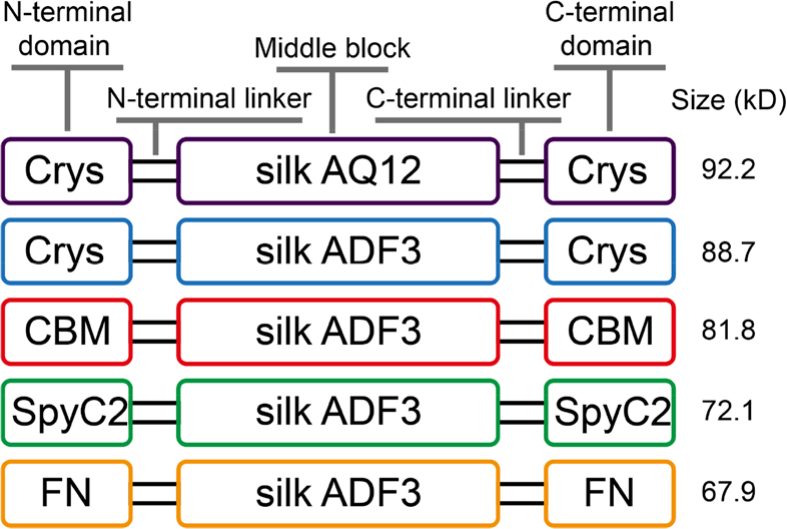
Schematic structures of the silk protein constructs used in this study.

Five different silk-like protein constructs were studied (Figure 1) each designed with a triblock structure: a highly repetitive sequence in the middle and globular terminal domains at both N and C termini. Two different mid-blocks were used: the major ampulla gland silk fibroin 3 (ADF3)^16,20^ from *A. diadematus* and an engineered version called AQ12^21^ that contains 12 repeats derived from ADF3. Four different globular proteins with different properties were used as terminal domains: the highly soluble gamma-crystallin D (Crys, 20.3 kDa) from nerve cells of *Homo sapiens*,^41^ the fibronectin III domain 10 (FN, 9.9 kDa, *H. sapiens*) that facilitates cell adhesion,^42^ the version 2 of SpyCatcher (SpyC2, 12 kDa) that forms isopeptide bonds with its counterpart (SpyTag),^43^ and the cellulose-binding module CBM3 (17 kDa) from *Ruminiclostridium thermocellum*. The protein constructs were named: Crys–ADF3–Crys, CBM–ADF3–CBM, FN–ADF3–FN, SpyC2–ADF3–SpyC2, and Crys–AQ12–Crys, as illustrated in Figure 1.

The cloning procedure has been described earlier.^16^ Sequences of the protein constructs can be found in the Supporting Information.

### 2.2 Protein Expression and Purification

The silk proteins were expressed in *Escherichia coli* BL21 strain. EnPresso media was used for protein expression according to the protocol of the manufacturer (EnPresso B 500, EnPresso GmbH). The cells were harvested after 24 h of induction (8000*g* for 10 min) and lysed by sonication (60% amplitude for 5 min). The proteins of interest were purified by nickel affinity chromatography ÄKTA-Pure and HisTrap FF crude columns (GE Healthcare Life Science). Proteins were then desalted using Econo-Pac 10DG gel filtration columns (Bio-Rad) and concentrated to >5 mg/mL using 30 kDa cutoff centrifugal concentrators with a poly(ether sulfone) membrane (Vivaspin, Sartorius). Finally, the protein solutions were frozen with liquid nitrogen and stored at −80 °C.

### 2.3 Silk Film Preparation

**Figure 2:**
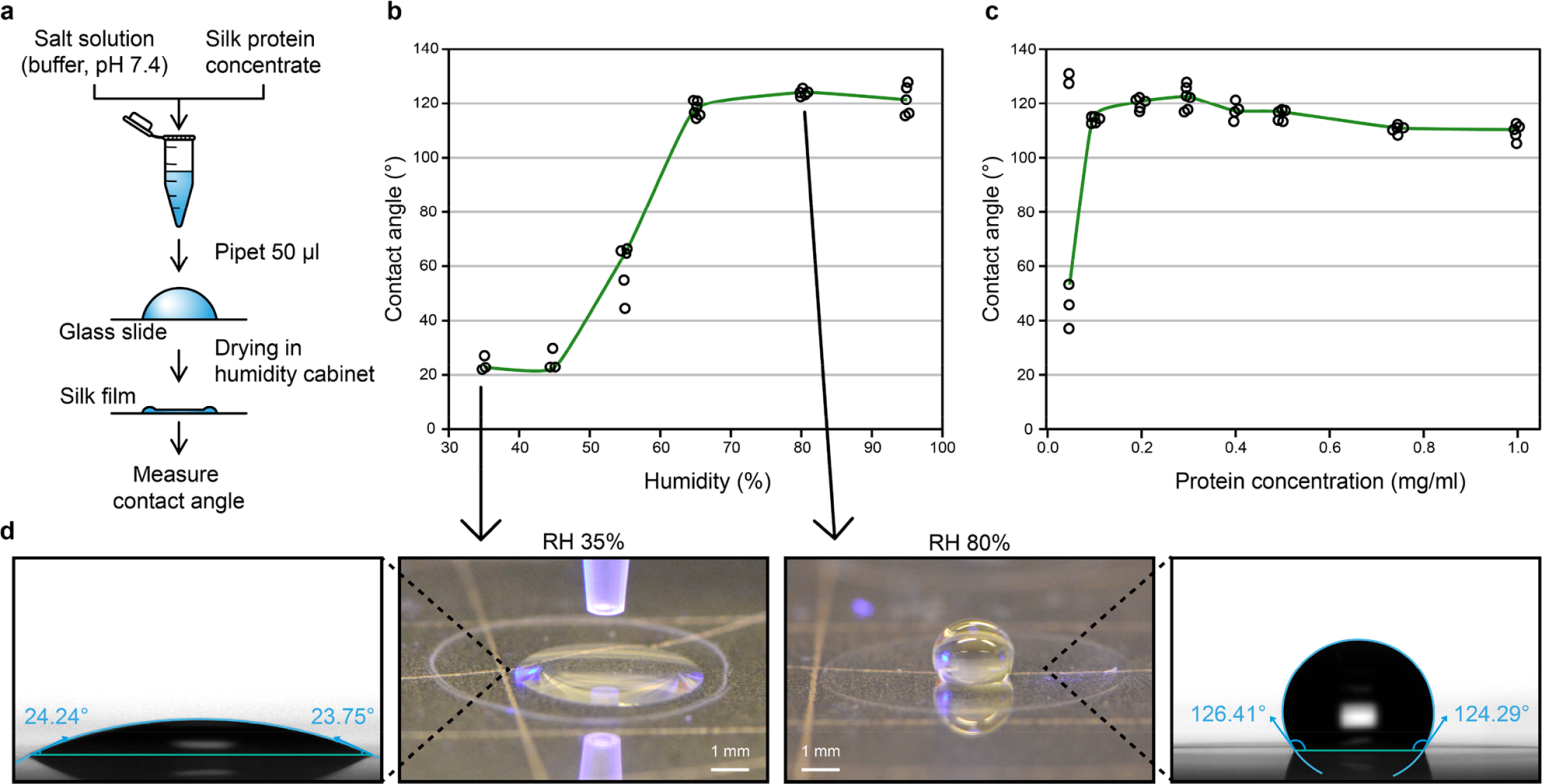
Preparation conditions have significant effects on the water contact angle of the silk films. (a) Preparation method for the silk films. (b) Effect of the RH during the silk film drying step to the contact angle (0.3 mg/mL silk Crys–ADF3–Crys in 0.5 mM Tris–HCl at pH 7.4). (c) Effect of the protein concentration on the contact angle (silk Crys–ADF3–Crys in 0.5 mM Tris–HCl at pH 7.4, prepared at 80% RH). (d) Images of films using the same protein but prepared at 35 and 80% RH with corresponding contact angles.

To achieve the desired protein concentration for casting the different films (0.02–1.0 mg/mL), the stock protein solution was thawed and then diluted with deionized water or an aqueous salt solution (0.1–8 mM, pH 7.4) according to the film being prepared. These consisted of tris(hydroxymethyl)aminomethane (Tris)–HCl, sodium acetate, sodium chloride, sodium phosphate, and sodium sulfate. Protein concentrations were determined with a spectrophotometer at 280 nm wavelength (NanoDrop, Thermo Fisher Scientific) using calculated extinction coefficients. To prepare films, 50 μL of protein solution was pipetted on a glass slide and placed in a humidity-controlled cabinet (35–95% relative humidity and 20 °C) and dried overnight. Generally, the films were circular with diameters within 7–9 mm and had slightly thicker edges. If the silk is assumed to spread evenly, 0.02, 0.3, and 1.0 mg/mL silk lead to approximately 2, 30, and 100 μg/cm^2^ silk, respectively. The preparation procedure is described in Figure 2a.

### 2.4 Optical Tensiometer

An optical tensiometer (Theta Flex, Biolin Scientific) was used to measure CAs of silk films. Static CAs were measured using a 4 μL drop of distilled water (unless stated otherwise), and the data capture time was 90 s at 3 frames per second. Dynamic CAs were measured with distilled water flow of 0.05 μL/s for both advancing and receding CAs. The advancing contact angle was measured until the volume of 20 μL was reached and then changed to measuring receding CA.

### 2.5 Scanning Electron Microscopy (SEM)

Silk films on glass slides were attached to an aluminum stub using carbon tape and then sputter-coated with 5 nm of gold/palladium. Images were taken with a Sigma VP electron microscope (Zeiss) using an acceleration voltage of 1.5 kV and a SE2 or an in-lens detector.

### 2.6 Circular Dichroism (CD)

CD spectroscopy was used to study the secondary structure of the silk proteins. Silk films were prepared on the side of a quartz crystal cuvette using 50 μL of 0.5 mg/mL silk protein. CD spectra between 180 and 260 nm were measured with a Jasco J-1500-150ST instrument (1 nm bandwidth and average of eight accumulations).

### 2.7 Atomic Force Microscopy (AFM)

The surface morphology and roughness of silk films were obtained using AFM (JPK NanoWizard IV XP-Bio-AFM). Films deposited on glass slides were analyzed using quantitative imaging mode. Imaging in air was performed with a triangular silicon nitride cantilever (tip radius 10 nm), a resonant frequency of 121 kHz, and a calibrated spring constant of 0.9 N/m. The surface topographical parameters were determined from AFM images using processing software (JPK Instruments).

### 2.8 X-ray Photoelectron Spectroscopy (XPS)

XPS was used to study changes in the top layer of the silk films. The measurements were performed with a Kratos AXIS Ultra DLD X-ray photoelectron spectrometer using a monochromatic Al Kα X-ray source (1486.7 eV) run at 100 W. A pass energy of 80 eV and a step size of 1.0 eV were used for survey spectra, while a pass energy of 20 eV and a step size of 0.1 eV were used for high-resolution spectra. Photoelectrons were collected at a 90° takeoff angle under ultrahigh vacuum conditions, with a base pressure typically below 1 × 10^–9^ Torr. The diameter of the beam spot from the X-ray was 1 mm, and the area of analysis for these measurements was 300 × 700 μm. Both survey and high-resolution spectra were collected from multiple spots on each sample surface, to check for homogeneity and surface charging effects. All acquired spectra were charge-corrected relative to the position of C–O bonding at 286.5 eV.

### 2.9 Statistics

For sample groups that were normally distributed and had similar variances, Student’s *t* test was used to study differences between the groups. In other cases, such as for non-normal sample group distribution, the more robust Wilcoxon signed-rank test was applied. One-way analysis of variance (ANOVA) was used to study differences between more than two sample groups. Data analysis was performed using the open-source software RStudio (R programming language). When results are reported in format “*a* ± *b*”, the first term (*a*) is the mean and the latter (*b*) is the standard deviation. In all graphs, a spline was fitted to go through median values of individual sample groups (geom_xspline in the ggalt package). When necessary for better visualization, individual data points were jittered along the *x* axis to separate them from each other.

## 3 Results and Discussion

Silk protein films were cast from an aqueous Crys–ADF3–Crys solution on glass slides (Figure 2a). The relative humidity (RH) during the silk film drying step was kept constant and varied between experiments from 35 to 95% RH. The wettability of silk films was studied with an optical tensiometer. Silk films prepared at below 45% RH showed high wettability (CA of <30°) (Figure 2b). When they were prepared at over 65% RH, the films had low wettability (CA of >120°). Typical images of CA measurements of films prepared at 35 and 80% RH are shown in Figure 2d. Different protein concentrations were also tested (Figure 2c). A low protein concentration (∼0.02 mg/mL) led to incomplete formation of films, which is likely the reason for the high variability in CA results. The highest CA average was obtained with a protein concentration of 0.3 mg/mL. Increasing the protein concentration further led to a slow decline in CAs. The stability of hydrophobic films (Crys–ADF3–Crys, prepared at 80% RH) was tested by measuring the static CA over extended periods. No spreading of the droplet was observed. Data for a 30 min period are shown in Figure S1 of the Supporting Information. Overnight experiments did also not show any spreading, but CA measurements become unfeasible as a result of droplet evaporation. Other support materials in addition to glass were also tested; the use of polystyrene, metal, and different hydrophilic and hydrophobic glass slides did not significantly impact CA values (Figure S2 of the Supporting Information). In a previous study, Wohlrab et al. reported that block copolymers, like silk, arrange differently depending upon the hydrophobicity of the support material.^5^ Such a phenomenon was not noted in our studies.

The films were further characterized by making a Zisman plot.^44^ For this, wetting of the hydrophobic silk film by a dilution series of isopropanol in water (0–32 vol %) was measured (Table S1 and Figure S3 of the Supporting Information). The critical surface tension was determined to be 22 mN/m. Further, testing the wetting properties with oils showed that hexadecane and silicon oil can fully wet the film.

The role of humidity and water during the formation of silk materials has been pointed out as a key parameter in previous reports.^45^ It serves as a plasticizer between the hydrophilic regions of silk, maintaining protein solubility. Previous studies have reached the conclusion that the rate of water removal, i.e., how quickly drying occurs, affects silk assembly. This effect could be based on the kinetics of the formation of intra- and intermolecular interactions.^45,46^ In our experiments, the use of different relative humidity levels during the drying of the silk film affected how fast the droplet of aqueous silk solution evaporated and formed the film. At 35% RH, the film formed in less than an hour; however, at 80% RH, it took overnight, and at 95% RH, it took around 30 h. On the basis of these data, however, it is not possible to distinguish the effects of overall drying time and RH on the CA of the film or if both together have a significant role. Previously, Mohammadi et al. showed that similar silk-like proteins can self-assemble at the water–air interface of a droplet in a manner that is both time- and RH-dependent.^47^ Furthermore, they noticed a significant difference in the β-sheet content between 40 and 80% RH,^47^ an observation consistent with our findings that the most dramatic change happens in the range of 45–65% RH. The effect of the protein concentration on CA indicates that a moderate amount of protein is better provided that the film contains enough protein for full surface coverage.

**Figure 3:**
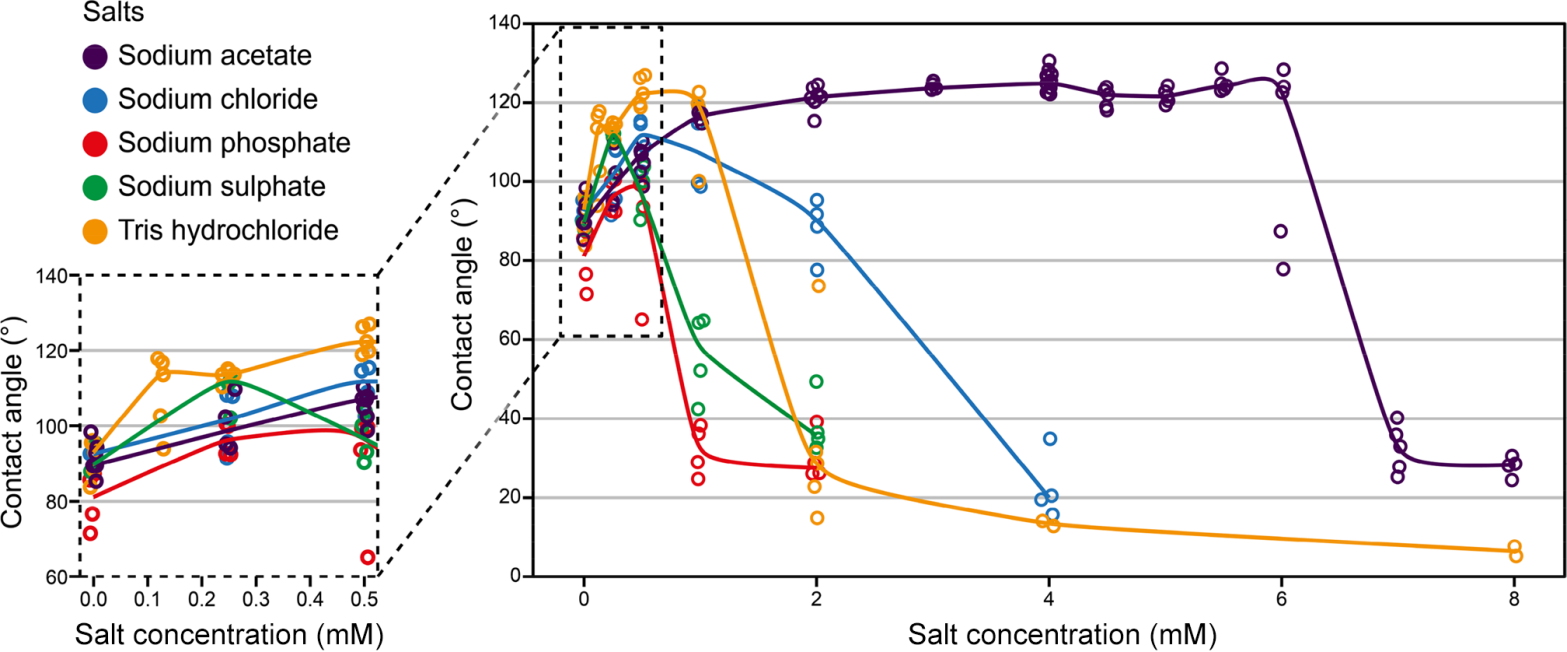
Effect of the salt on contact angles of silk films. Films were prepared from 0.3 mg/mL silk Crys–ADF3–Crys in various salt concentrations at 80% RH. In all cases, the addition of a small amount of salt resulted in an increases of the contact angle.

Next, the effect of salt was tested by mixing the silk protein with 0–8 mM of various salts (Tris–HCl, sodium acetate, sodium chloride, sodium phosphate, and sodium sulfate, all adjusted to pH 7.4). The results show that, in pure water, the silk films have CAs of 89.6 ± 6.1° (Figure 3). An addition of any salt increased the CA of the protein films, although to different extents depending upon the type of salt. Each salt had a threshold concentration after which the CA decreased rapidly. Both Tris–HCl and sodium acetate led to an increase of the CA by more than 30° to reach over 120°. Silk films with 0.5 mM Tris–HCl achieved a CA of 122.8 ± 3.7°, and silk films with 4 mM sodium acetate achieved a CA of 125.3° ± 2.6°. Small amounts of sodium chloride (0.5 mM), sodium phosphate (0.5 mM), and sodium sulfate (0.25 mM) increased the CA by around 20°. Interestingly and unlike other salts that were used here, a broad concentration range of sodium acetate (2–6 mM) resulted in CAs that were over 120°.

Salts are known to interact with proteins by salting-in (solubilizing) or salting-out (aggregating and structural formation). Certain salts are more efficient in salting-out proteins than others, and the ability to do this is described by the Hofmeister series. The anions used in this study can therefore be ranked in terms of efficiency in salting-out: sulfate > phosphate > acetate > chloride (Table 1).^45^ The cations used in this study were sodium and Tris, but Tris is not typically listed in the Hofmeister series. Tris is known to interact with proteins and stabilize them.^48^ Previously, it has been found that sodium acetate, sodium phosphate, and sodium sulfate induce both LLPS and the transition of secondary structures from disordered to β-sheet-rich structures in the order: sodium sulfate > sodium phosphate > sodium acetate (Table 1).^18^ This order follows the Hofmeister series.

**Table 1:**
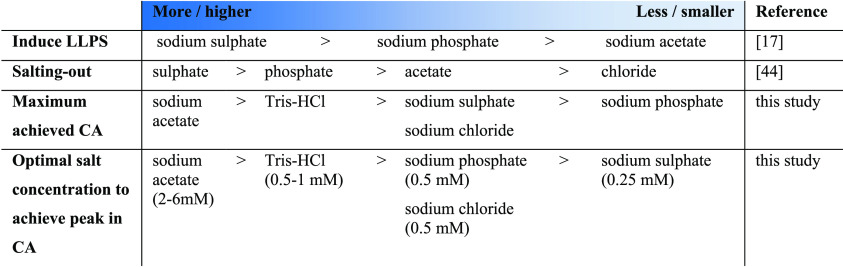
Comparison of Salts

Ranking the salts from most to least significant increase of CA gives sodium acetate > Tris–hydrochloride > sodium sulfate = sodium chloride > sodium phosphate (Table 1). There does not seem to be any correlation between the maximum CA and the Hofmeister series. Sodium sulfate should have the highest ability to salt-out, and sodium chloride should have the least; however, they increased the CA to the same extent. On the other hand, the strongest declines in CA were caused by sodium sulfate and sodium phosphate, which are the most efficient in salting-out proteins.^45^ The salt with the least ability to salt-out, sodium chloride, caused a slow decline in CA with an increasing salt concentration and did not increase the CA above 110°. This would indicate that, while strong salting-out is harmful in forming the hydrophobic surfaces, a certain level is still necessary. It should, however, be noted that interpreting the Hofmeister series might not be as straightforward as often presented. For instance, anions might have complex protein-specific interactions that do not follow the series.^49^

The concentration of salt also played an important role, with each salt exhibiting a unique response profile. Ranking by the optimal salt concentration to achieve the peak in CA results in the following list: sodium sulfate (0.25 mM) < sodium chloride = sodium phosphate (0.5 mM) < Tris–hydrochloride (0.5–1.0 mM) < sodium acetate (2–6 mM) (Table 1). Again, it seems that salts with moderate ability to salt-out are tolerated at higher concentrations. In addition, the order of sodium acetate, sodium phosphate, and sodium sulfate corresponds to their ability to induce LLPS and transition to β-sheet structures, with sodium acetate being the weakest out of the three salts.^18^ This indicates that strong LLPS and/or transition to β-sheet structures could be disadvantageous. The weak effect of sodium acetate is more likely to lead to high CA films. At the optimal salt concentration, the mass ratio between salt and silk protein varied notably: sodium chloride, sodium sulfate, and sodium phosphate were 0.1–0.2 times the mass of silk, and Tris–HCl and sodium acetate were 0.25–0.5 and 0.5–1.6 times the mass of silk, respectively.

**Figure 4:**
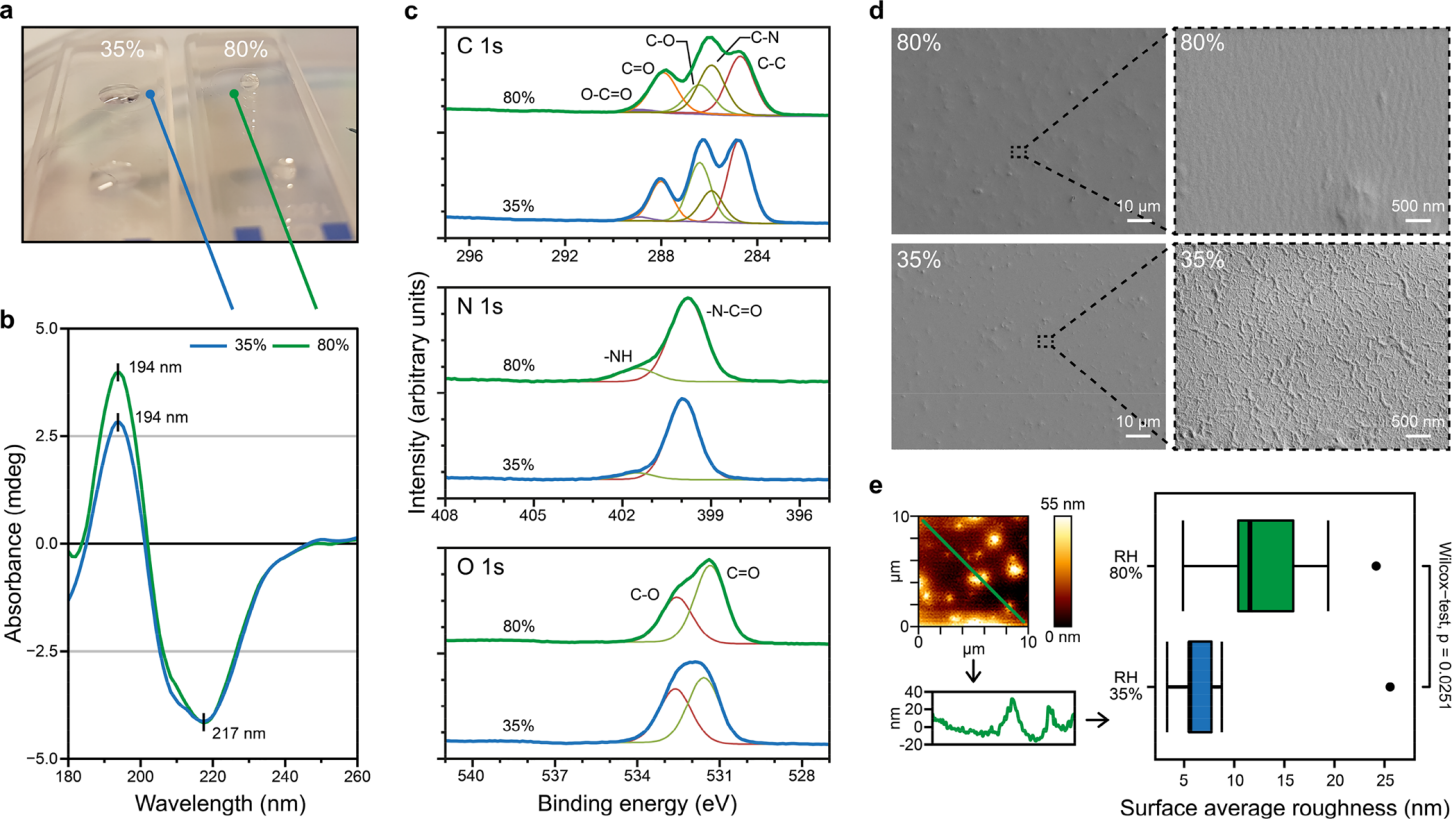
Structure of silk films. (a) Quartz crystal cuvettes coated with silk and prepared in 35 and 80% RH (0.5 mg/mL Crys–ADF3–Crys in 0.5 mM Tris–HCl). A water droplet was placed on the film after CD measurement to demonstrate the wetting behavior of the film. (b) CD of the silk films showing identical peak locations for both films. Peak locations of 194 and 217 nm suggest a β-sheet-rich structure. (c) C 1s, N 1s, and O 1s high-resolution XPS spectra comparing 35 and 80% RH films. The spectra reveal differences in the structural composition of the top part of the film. (d) SEM images of silk films prepared at 35 and 80% RH (0.3 mg/mL Crys–ADF3–Crys in 0.5 mM Tris–HCl). (e) Surface roughness of the same silk films, calculated from AFM topography images.

Next, we examined if the secondary structural features of the proteins could explain the intrinsic hydrophobicity of the films. The most hydrophobic regions in silk protein are the alanine (−CH_3_ side chain)-rich blocks. The way in which the protein chains are arranged both intra- and intermolecularly can impact the extent to which alanine regions are exposed at the surface of the film. For CD and XPS, we used 0.5 mg/mL Crys–ADF3–Crys in 0.5 mM Tris–HCl prepared films at either 30 or 85% RH. CD was used to study differences in the protein secondary structure between the two sets of films. The CD was measured on films coated on the side of quartz cuvettes (Figure 4a). Droplets of water were placed afterward on the films to confirm their hydrophobicity. The CD spectra showed only minor differences in the secondary structures of the proteins between the films prepared at 35 and 80% RH; both have a positive peak at 194 nm and a negative peak at 217 nm, which suggests a β-sheet-rich structure. The similarity of spectra does not exclude the possibility for minor structural differences as a result of the relative insensitivity of CD. For example, the difference in peak heights may indicate differences in the ratio of different secondary structures (Figure 4b).^50^ The composition of the films prepared at 35 and 80% RH was further examined with XPS. The likelihood of photoelectron emission decreases rapidly with increasing depth in the sample, which makes XPS a very surface-sensitive technique, with a total maximum probing depth of around 5–10 nm. From the individual C 1s, O 1s, and N 1s high-resolution spectra (Figure 4c and Figures S6–S8 of the Supporting Information), it is evident that the structural composition of the top part of the film is different depending upon the preparation method. In this upper layer, the 80% RH (high CA) films were found to contain relatively more nitrogen, C–N, C=O, and NH-bonding environments as well as chloride salts compared to the 35% RH (low CA) films (Tables S2–S5 of the Supporting Information). On the other hand, the low CA films contained more carbon, oxygen, C–C, C–O, O–C=O, −N–C=O, −NOH, and C–OH bonding environments. We next analyzed the side-chain composition of the two main parts of the protein, crystallin and ADF3, excluding contributions from peptide bonds. The crystallin part contains more O–C=O and C–OH bonds than the ADF3 region. The results therefore show that, in high CA 80% RH films, the ADF3 part of the protein is relatively more abundant closer to the surface than in the low CA 35% RH films.

Next, we studied whether there are differences in the topographical structures that could contribute to the hydrophobicity of the films. The topographies of silk films prepared at RH of 80 and 35% were studied with SEM and AFM. At first glance, the SEM images show that both films contain protein aggregates seemingly at similar densities (Figure 4d), approximately 200 aggregates/mm^2^. However, the surface between the aggregates differs: 35% RH films have a wrinkled, randomly orientated surface, while 80% RH films have a smoother, wavy surface structure. In contrast, surface average roughness calculated from AFM images (Figure 4e and Figures S9 and S10 of the Supporting Information) show that 80% RH films have higher surface roughness (*p* < 0.05). However, the surface roughness is dominated by protein aggregates with a height up to 500 nm and not by the small structures in between with a height under 10 nm. Probing the stiffness of the two films with the AFM tip showed no significant difference (Figure S11 of the Supporting Information).

**Figure 5:**
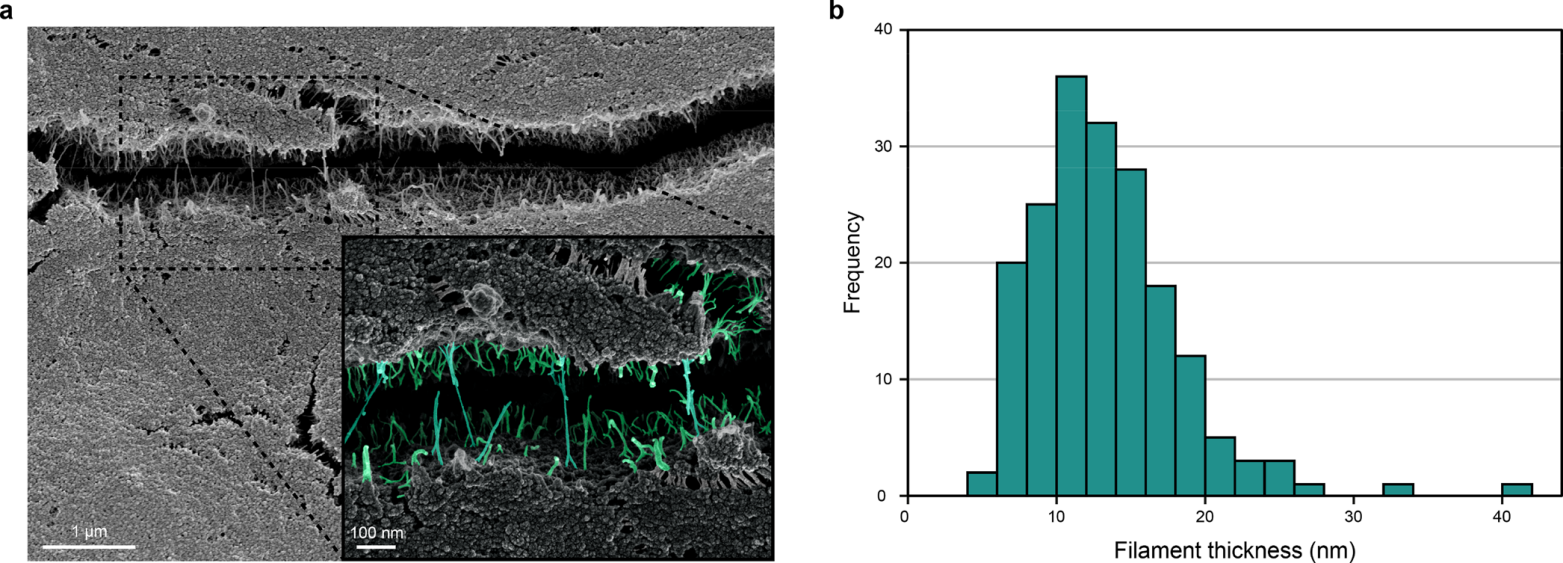
Nanosized silk filaments bridging over a crack in a silk film, indicating a presence of assembled structures of the silk proteins in the film. (a) SEM image of an 80% RH silk film that shows silk filaments bridging over a crack. The zoomed image shows filaments highlighted by false coloring. (b) Histogram of the silk filament thickness in the close-up SEM image. Only colored filaments were included. The average filament size was 13.2 ± 5.0 nm.

While testing different support materials, films from Crys–ADF3–Crys were prepared on rough aluminum SEM stubs, which caused several cracks on the film during the drying step at both 35 and 80% RH. This allowed for the further study of the internal structure of the silk films by SEM. Both films showed nanosized silk filaments bridging over the cracks and an unusually rough film surface (80% RH film in Figure 5a). The 35% RH films had shorter and fewer filaments (Figure S12 of the Supporting Information), but during drying, the silk solution spread on the whole aluminum stub (diameter of 12.5 mm), while at 80% RH, it stayed within 6–7 mm diameter, making direct comparisons difficult. The visible filaments in the higher magnification image were selected with a graphical editor and colored with an overlay (Figure 5a). An image processing program was used to measure the average thicknesses of the filaments (Figure 5b). Without considering the influence of Au/Pd coating, the filaments were on average 13.2 ± 5.0 nm. On the basis of these images, the silk protein undergoes molecular self-assembly into thin filaments during film formation. Similar assemblies have been reported previously;^16,51^ however, unlike the previously reported structures, the structures observed here were very small. To illustrate, if one silk protein including its water shell is assumed to have a thickness of 2 nm,^52^ with a very rough estimation, a 13 nm thick filament would contain 6–7 silk proteins side by side.

Considered together, the characterization of the silk film surface structure shows both nano- and microlevel structural features. On the basis of the SEM and AFM data, both features seem to be affected by the RH during film preparation. However, the structural features do not explain the difference in hydrophobicity between the films from high- and low-humidity preparation methods (CA of >120° and <25°, respectively).^38,40^ The average surface roughness of the silk films was similar to what has been reported before on silk films by Wohlrab et al.^5^ In comparison to other published reports on non-silk films with high hydrophobicity, the average surface roughness of the 80% RH film was low, 13 nm versus 190 nm (CA of >160°),^33^ and the spacing between the microstructures was sparce in relation to the size of the microstructures. The microstructures in the silk films had average heights of ∼200 nm and spacings of ∼5 μm compared to 200 and 100 nm for a film with CA of >160°,^40^ 5 and 5 μm for a film with CA of >160°,^53^ 10–50 and 10–30 μm for a film with CA of >160°,^39^ and 10 and 10 μm for a film with CA of >150°.^54^ It is noteworthy that, even with the lack of defined topography, the silk films displayed CAs up to 130°. It remains unclear what was the role of the silk film topography, but very likely, it is the intrinsic hydrophobicity and not topography that leads to the high CA in the films.

**Figure 6:**
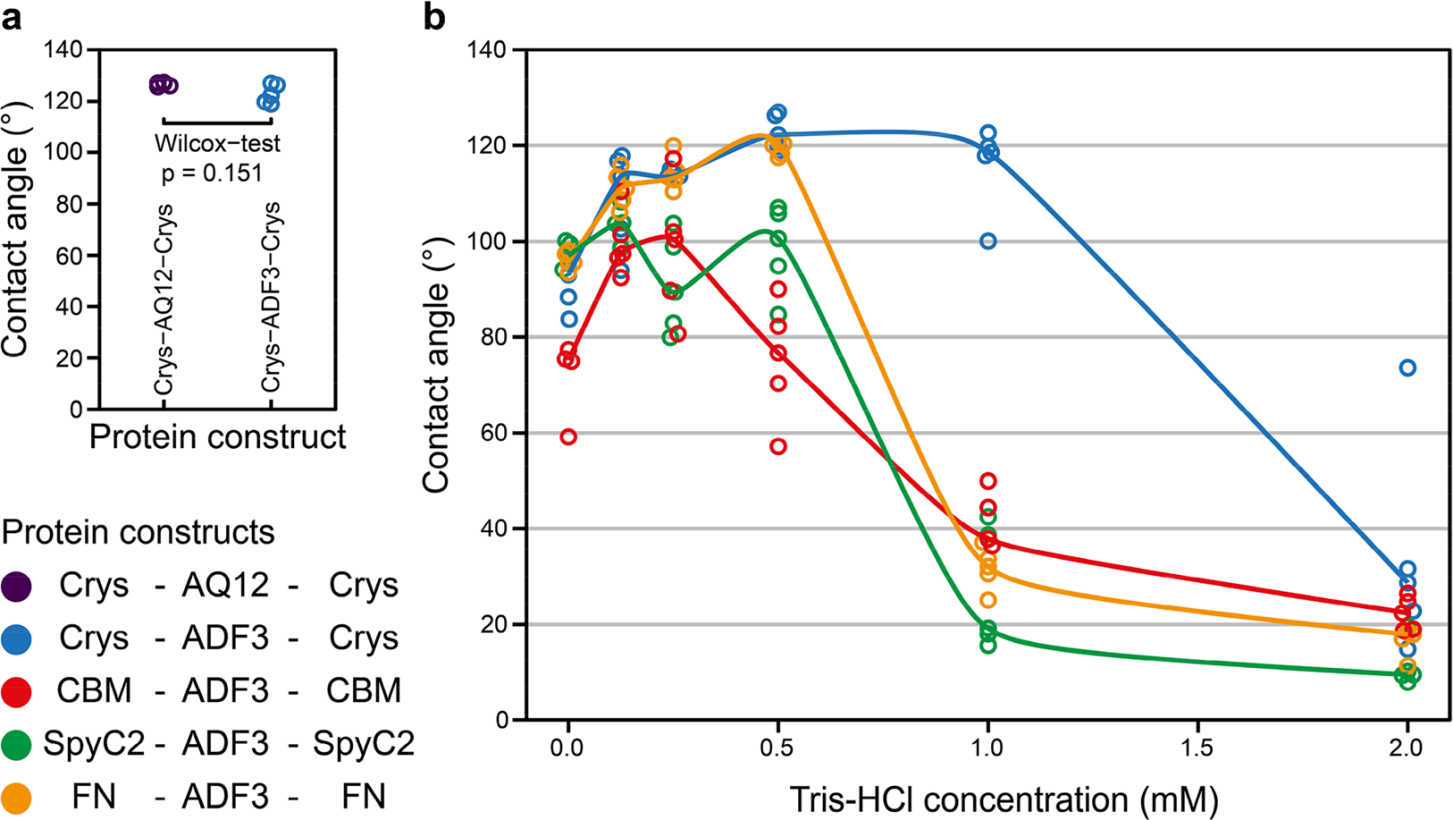
Contact angles of different silk constructs in 0–2 mM Tris–HCl at pH 7.4, prepared at 80% RH. (a) Effect of the mid-block in protein constructs on the contact angle (0.3 mg/mL protein in 0.5 mM Tris–HCl, prepared at 80% RH) shows no significant difference (*p* > 0.05). (b) Effect of the terminal domain in protein constructs to the contact angle in 0–2 mM Tris–HCl (0.3 mg/mL protein in 0.5 mM Tris–HCl, with the exception of FN–ADF3–FN, which is 0.1 mg/mL).

Different protein constructs were tested to understand the contribution of different terminal domains and mid-blocks (Figure 6). Using a set of different terminal domains, we could on a general level probe of how sensitive the silk blocks were to their immediate physicochemical environment. The terminal domains represent a selection of folded globular proteins of roughly the same size as the natural N- and C-terminal domains, but we did not have any *a priori* information that they would affect hydrophobicity in any specific way. The reason for analyzing this set was to screen for the sensitivity of the constructs for variations in the architecture. The constructs that were tested are Crys–AQ12–Crys, Crys–ADF3–Crys, CBM–ADF3–CBM, SpyC2–ADF3–SpyC2, and FN–ADF3–FN. The effect of different mid-blocks, ADF3 and its engineered version AQ12, were tested by preparing 0.3 mg/mL of the silks Crys–ADF3–Crys and Crys–AQ12–Crys in 0.5 mM Tris–HCl and prepared into silk films as previously at 80% RH. Both films achieved high CAs (Figure 6a) with no significant difference (*p* > 0.05), indicating that the two mid-blocks have identical behavior. To exclude the possibility that Crys domains alone would show hydrophobicity, films were also prepared using purified Crys protein with various Tris–HCl concentrations. These films of Crys proteins were all highly hydrophilic, with CAs under 20° (Figure S13 of the Supporting Information). Next, the effect of replacing Crys with other terminal domains while keeping the same ADF3 mid-block was tested. A set of films with the variants Crys–ADF3–Crys, CBM–ADF3–CBM, SpyC2–ADF3–SpyC2, and FN–ADF3–FN were prepared. A protein concentration of 0.3 mg/mL was used, and the Tris–HCl concentrations varied from 0 to 2 mM. The films were then dried at 80% RH. The only exception to this was FN–ADF3–FN, where 0.1 mg/mL protein was used to prepare the films, because 0.3 mg/mL caused high variation in CAs (results not shown).

Without salt, the CA was around 90.0 ± 10.9° for all of the silk constructs, except CBM–ADF3–CBM, which had a lower CA of 71.7° on average (Figure 6b). With Crys–ADF3–Crys, CBM–ADF3–CBM, and FN–ADF3–FN, a small amount of Tris–HCl increased the CA by 20–30°. At 0.5 mM Tris–HCl, FN–ADF3–FN achieved a CA of 119.4 ± 1.5°, which was on the same level as Crys–ADF3–Crys. While the CA of CBM–ADF3–CBM did increase by adding Tris–HCl, it only reached a CA of 100°. With SpyC2–ADF3–SpyC2, Tris–HCl showed no difference compared to pure water. From these results, we can conclude that the wetting properties of the Crys–ADF3–Crys film are not critically dependent upon the terminal domain Crys, and replacing it with other terminal domains can lead to similar behavior. However, overall, Crys–ADF3–Crys did give the highest CAs over a broader range of Tris–HCl concentrations compared to other constructs.

**Figure 7:**
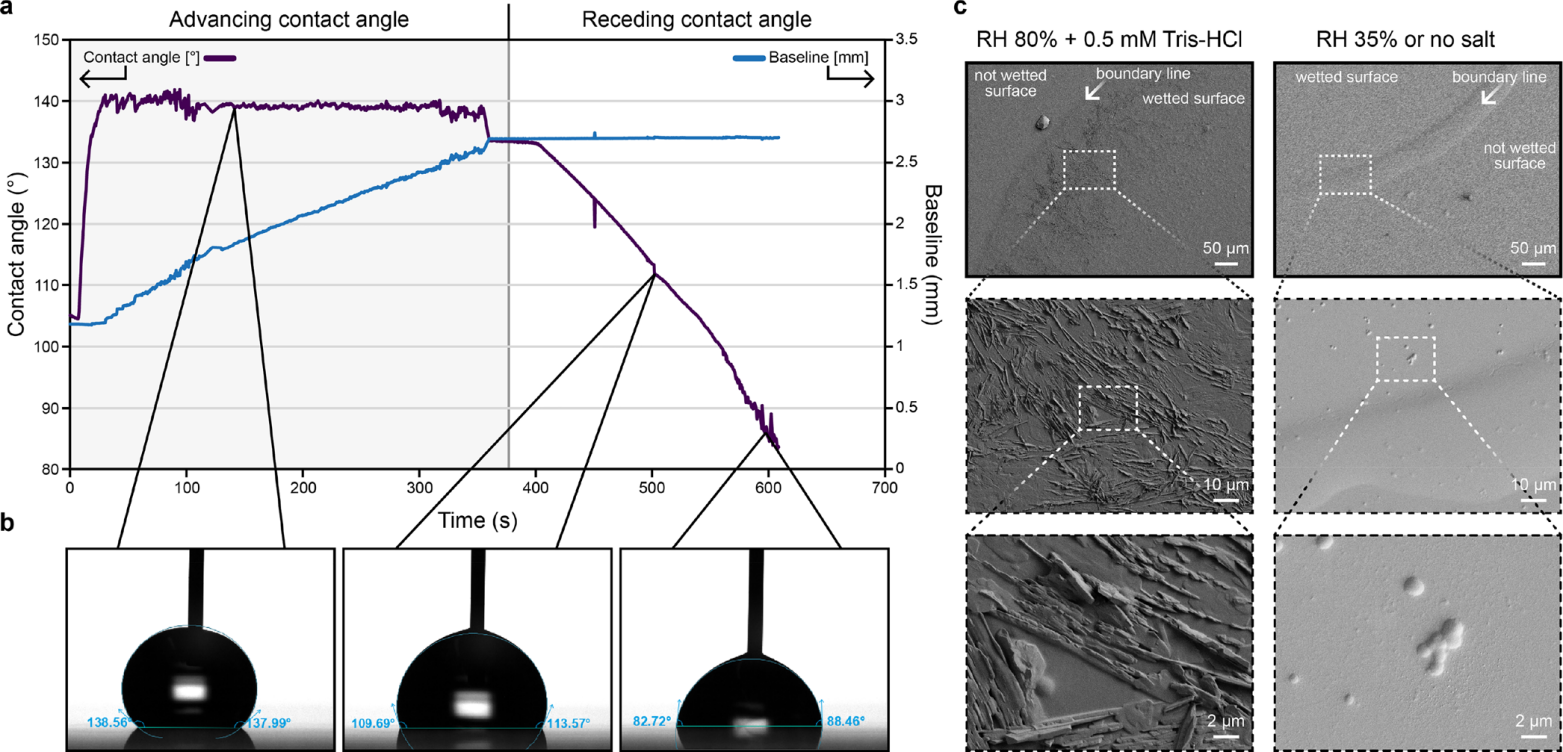
(a) Dynamic contact angle measurement of a silk film (0.5 mg/mL Crys–ADF3–Crys in 0.5 mM Tris–HCl, dried at 80% RH). The behavior during advancing contact angle measurement was normal, but the receding contact angle showed that wetting by water disturbs the film, causing water to adhere to it. (b) Images of the dynamic contact angle measurement, showing the high contact angle and how the baseline stopped decreasing during receding contact angle measurement. (c) SEM images of silk films that were first wetted with a 4 μL droplet of water, dried, and then imaged (silk Crys–ADF3–Crys). The films that initially were wetting-resistant showed crystal-like structures after being wetted.

Dynamic contact angles of high CA (120°) films prepared from Crys–ADF3–Crys at 80% RH were studied to further understand their properties. A small droplet was placed on the silk film, and more water was slowly injected, causing the droplet to expand. When a surface is homogeneous, the CA of the advancing droplet (ACA) should stay constant, while the contact area (baseline) between the droplet and surface increases steadily,^55^ as seen in Figure 7a. The ACA of the silk film was 140°. Then, the droplet was slowly aspirated to observe the receding contact angle (RCA). Typically for homogeneous water-stable films, the droplet should have a fairly constant RCA and steady decline in the baseline.^55^ However, in the case of the silk films, the baseline stayed constant while the RCA declined, indicating that water started to adhere to the surface, and thus, no receding contact angle could be obtained.

To try to explain the behavior of the silk films in the receding contact angle measurements, a droplet of water was placed on a high CA film (80% RH with 0.5 mM Tris–HCl), a medium CA (∼90°) film (80% RH with 0 mM Tris–HCl), and a low CA (<20°) film (35% RH with 0.5 mM Tris–HCl). The water was then allowed to dry at room temperature. All films had a very slight marking visible to the naked eye left from the wetting, showing changes in the surface structure (results not shown). Under SEM, the high CA films contained crystal-like structures in the region, which had been wetted. Such crystal-like structures were not present in the films prior to wetting (Figure 7c and Figures S14 and S15 of the Supporting Information). The medium (no salt) and low CA (35% RH) films both showed no change between the non-wetted and wetted regions. There was however a thick boundary between the two regions, which was similar to the outer edge of the films. After drying, the high CA films showed a clear decrease in CA from >120° to around 90° (Figure S16 of the Supporting Information). Wetted high CA films were further analyzed with XPS, which showed an increase of chlorine, C–O-bonded carbon, and nitrogen in the form of amines on the wetted surface compared to the non-wetted surface (Tables S6–S10 and Figures S17–S22 of the Supporting Information). The results point toward an increase of Tris–HCl and a decrease of proteins on the top layer (up to 10 nm depth) of the film. Thus, the crystal-like structures were likely residues of Tris–HCl. The films were disrupted by water, which led to a rearrangement of salts and proteins in the film. In a previous publication, a similar phenomenon was observed with a coating of 50:50 formamide/poly(lactic/glycolic acid), where ACA was stable at 60° but RCA was declining steadily.^56^ They stated that, for certain systems, the energy barrier for the liquid to recede is too high to overcome.^56^ It has been shown previously that materials made from recombinant silk can be water-soluble unless post-treated^12,14,15,24,27^ and that salt interactions are an important part of the silk assembly.^18,57,58^ We attempted post-treatment with methanol and heating, but this was unsuccessful and reduced the CA from ∼120° to ∼90° (Figure S23 of the Supporting Information).

## 4 Conclusion

In this study, we developed a method for the preparation of thin silk films with hydrophobic properties. Under carefully controlled conditions, triblock fusion proteins of both AQ12 and ADF3 can assemble in a way in which their hydrophobic side chains are highly exposed at the outside surface of the film. The hydrophobic properties of the films were only observed when a certain amount of salt was mixed with the silk protein solution. For the formation of hydrophobic films, a careful control of conditions and concentrations was necessary.

In optimized silk films, static CAs up to 120–130° and advancing CA of 140° were achieved. It is generally thought that hydrophobic flat films without the contribution of topographic features can achieve a maximum contact angle of around 120°,^33−38^ which is slightly lower than the CAs achieved by the silk films here. Some micro- and nanoscale structures were observed at the surface of the silk films. While the role of the surface structure in conferring high CAs to the silk films remains unresolved, it likely plays a minor role. Instead, it is likely that the properties are due to the intrinsic hydrophobicity of the silk proteins.

The silk part of the proteins contain 12 repeats of each six alanine residues. The assembly pathway toward conformations that expose them at the outer layer is critical. The pathway is sensitive to external conditions, as shown by the strong dependence upon the surrounding humidity, salts, and protein concentration itself. On the basis of our XPS data, we suggest that, in the high CA films, the proteins are somehow embedded or supported by the salt ions and terminal domains while exposing the hydrophobic segments of the silk part. The nature of the terminal domains affected how different variants behaved. This is expected because the terminal domains would be embedded below the exposed silk parts, and we can anticipate that terminal domains interact with salts differently. The tendency of proteins to form nanofibrils may be related to the structural assembly, leading to hydrophobicity, but we lack data on how exactly this would occur. The nanofibers could also be an alternative assembly route within the bulk of the films and not leading to exposed hydrophobicity.

The fact that proteins with AQ12 and ADF3 mid-blocks behaved identically in the experiments is interesting. The AQ12 version is a synthetically designed version having identical repeat units, whereas the ADF3 version shows more irregularity on the details of the repeats. The irregularity does not apparently lead to much difference in how the protein functions.

For material applications, polymers that assemble into hydrophobic coatings are in high demand. New solutions are needed because the fluorochemicals currently used to produce these coatings are likely to be phased-out because of environmental concerns. As such, our work does not provide a solution for durable coatings as a result of their instability after wetting. Nonetheless, our work can be seen as a proof of concept, because very high contact angles were achieved. To increase durability, suitable cross-linking, or binding through, for example, specially engineered terminal domains could provide solutions. Further engineering of the protein terminal domains could provide many options for this. On the other hand, the restructuring and loss of hydrophobicity after water exposure is unique and may, of course, inspire new types of uses, where it could be desirable that water contact changes the surface properties. Such a robust marker could be used to trace possible water exposure of surfaces, to direct water flow, or to function as a water-sensitive authenticity indicator.

The high hydrophobicity and amphiphilicity of the silk molecules suggest that hydrophobic interactions could be important in their natural assembly into and properties as fibers. The role of hydrophobic alanine stretches could form inter- or intramolecular clusters during assembly. The spinning duct of the spider does not provide hydrophobic interfaces, but structural arrangement of the polymer chains during assembly can provide interchain hydrophobic environments. Learning the nature of these and how they participate in assembly can show a path for silk assembly into a wide range of materials.

## Supporting Information Available

Static contact angle of a hydrophobic silk film over a longer period of time (Figure S1), contact angle of silk films coated on different surfaces (Figure S2), Zisman plot of the hydrophobic silk film (Figure S3), illustration of the silk proteins on the film surface (Figure S4), XPS survey spectra of silk films prepared at 35 and 80% RH (Figure S5), XPS high-resolution C 1s, N 1s, and O 1s spectra of silk films prepared at 35 and 80% RH (Figures S6–S8), AFM topography of silk films prepared at 80% RH (Figure S9), AFM topography of silk films prepared at 35% RH (Figure S10), AFM Young’s modulus of silk films prepared at 35 and 80% RH (Figure S11), cracked silk films on an aluminum stub prepared at 35% RH (Figure S12), contact angle of bovine serum albumin (BSA) and crystallin films prepared at 80% RH (Figure S13), crystal-like structures in the silk film after wetting by water and drying (Figure S14), crystal-like structures in different patterns in the silk film after wetting by water and drying (Figure S15), changes in the contact angle of the silk film after wetting by water and drying (Figure S16), XPS survey spectra of high CA silk films (wetted and dry) and a glass slide (Figure S17), XPS high-resolution C 1s, N 1s, O 1s, Cl 2p, and Na 1s spectra of high CA silk films (wetted and dry) and a glass slide (Figures S18–S22), effect of methanol post-treatment on the contact angle of the silk film (Figure S23), surface tension of the isopropanol–water dilution series (Table S1), relative concentration of elements in all samples (35 and 80% RH films) (Table S2), relative amounts of the different components of carbon (35 and 80% RH films) (Table S3), relative amounts of the different components of nitrogen (35 and 80% RH films) (Table S4), relative amounts of the different components of oxygen (35 and 80% RH films) (Table S5), relative concentrations of elements in all samples (wetted and dry films) (Table S6), relative amounts of the different components of carbon (wetted and dry films) (Table S7), relative amounts of the different components of nitrogen (wetted and dry films) (Table S8), relative amounts of the different components of oxygen (wetted and dry films) (Table S9), and relative amounts of the different components of sodium (wetted and dry films) (Table S10) (PDF)

## Abbrevations Used

ACA: advancing contact angle
ADF3: major ampulla gland silk fibroin 3
AFM: atomic force microscopy
CA: contact angle
CBM: cellulose-binding module
CD: circular dichroism
Crys: gamma-crystallin D
FN: fibronectin III domain 10
LLPS: liquid–liquid phase separation
RCA: receding contact angle
RH: relative humidity
SD: standard deviation
SEM: scanning electron microscopy
SpyC2: SpyCatcher version 2
Tris: tris(hydroxymethyl)aminomethane
XPS: X-ray photoelectron spectroscopy
