# Supplementary material for: Highly Hydrophobic Films of Engineered Silk Proteins by a Simple Deposition Method
Source: Langmuir. 2023 Mar 16;39(12):4370–81. doi: 10.1021/acs.langmuir.2c03442 (PMC10061925; doi:10.1021/acs.langmuir.2c03442)
Supplement: Supplementary file 1 — la2c03442_si_001.pdf [file la2c03442_si_001.pdf]

Supporting Information

Highly hydrophobic films of engineered silk proteins by a simple deposition method

Teemu Välisalmi[1,3], Nelmary Roas-Escalona[1,3], Kristoffer Meinander[1,3], Pezhman Mohammadi[2], Markus B. Linder[1,3*]*

1: Department of Bioproducts and Biosystems, School of Chemical Engineering, Aalto University, FI-00076 Aalto, Finland

2: VTT Technical Research Centre of Finland Ltd. VTT, FI-02044 Espoo, Finland

3: Centre of Excellence in Life-Inspired Hybrid Materials (LIBER), Aalto University, P.O. Box 16100, FI-00076 Aalto, Finland

*Corresponding authors

Email: teemu.valisalmi@aalto.fi and markus.linder@aalto.fi

Crys-ADF3-Crys sequence

Structure: Terminal domain (Crys)-*Linker*-Mid-block (ADF3)-*Linker*-Terminal domain (Crys)-*His-tag*

MGKITLYEDRGFQGRHYECSSDHPNLQPYLSRCNSARVDSGCWMLYEQPNY
SGLQYFLRRGDYADHQQWMGLSDSVRSCRLIPHSGSHRIRLYEREDYRGQM

IEFTEDCSCLQDRFRFNEIHSLNVLEGSWVLYELSNYRGRQYLLMPGDYRRY

QDWGATNARVGSLRRVIDFS*PSASASASAGASAAASAGAGAGA*GPGQQGPGQQ

GPGQQGPYGPGASAAAAAAGGYGPGSGQQGPSQQGPGQQGPGGQGPYGPG

ASAAAAAAGGYGPGSGQQGPGGQGPYGPGSSAAAAAAGGNGPGSGQQGA

GQQGPGQQGPGGSAAAAAAGGYGPGSGQQGPGQQGPGGQGPYGPGASAA

AAAAGGYGPGSGQGPGQQGPGGQGPYGPGASAAAAAAGGYGPGSGQQGP

GQQGPGQQGPGGQGPYGPGASAAAAAAGGYGPGYGQQGPGQQGPGGQGP

YGPGASAASAASGGYGPGSGQQGPGQQGPGGQGPYGPGASAAAAAAGGY

GPGSGQQGPGQQGPGQQGPGQQGPGGQGPYGPGASAAAAAAGGYGPGSG

QQGPGQQGPGQQGPGQQGPGQQGPGQQGPGQQGPGQQGPGQQGPGGQGA

YGPGASAAAGAAGGYGPGSGQQGPGQQGPGQQGPGQQGPGQQGPGQQGP

GQQGPGQQGPYGPGASAAAAAAGGYGPGSGQQGPGQQGPGQQGPGGQ*ASA*

*SASAAASAASTVANSSS*KITLYEDRGFQGRHYECSSDHPNLQPYLSRCNSARVD

SGCWMLYEQPNYSGLQYFLRRGDYADHQQWMGLSDSVRSCRLIPHSGSHRI

RLYEREDYRGQMIEFTEDCSCLQDRFRFNEIHSLNVLEGSWVLYELSNYRGR

QYLLMPGDYRRYQDWGATNARVGSLRRVIDFS*LEHHHHHH*

CBM-ADF3-CBM sequence

Structure: Terminal domain (CBM)-*Linker*-Mid-block (ADF3)-*Linker*-Terminal domain (CBM)-*His-tag*

MGNLKVEFYNSNPSDTTNSINPQFKVTNTGSSAIDLSKLTLRYYYTVDGQKD

QTFWCDHAAIIGSNGSYNGITSNVKGTFVKMSSSTNNADTYLEISFTGGTLEP

GAHVQIQGRFAKNDWSNYTQSNDYSFKSASQFVEWDQVTAYLNGVLVWG

KEP*SASASASAGASAAASAGAGAGA*GPGQQGPGQQGPGQQGPYGPGASAAAA

AAGGYGPGSGQQGPSQQGPGQQGPGGQGPYGPGASAAAAAAGGYGPGSG
QQGPGGQGPYGPGSSAAAAAAGGNGPGSGQQGAGQQGPGQQGPGGSAAA
AAAGGYGPGSGQQGPGQQGPGGQGPYGPGASAAAAAAGGYGPGSGQGPG
QQGPGGQGPYGPGASAAAAAAGGYGPGSGQQGPGQQGPGQQGPGGQGPY
GPGASAAAAAAGGYGPGYGQQGPGQQGPGGQGPYGPGASAASAASGGYG
PGSGQQGPGQQGPGGQGPYGPGASAAAAAAGGYGPGSGQQGPGQQGPGQ
QGPGQQGPGGQGPYGPGASAAAAAAGGYGPGSGQQGPGQQGPGQQGPGQ
QGPGQQGPGQQGPGQQGPGQQGPGQQGPGGQGAYGPGASAAAGAAGGYG
PGSGQQGPGQQGPGQQGPGQQGPGQQGPGQQGPGQQGPGQQGPYGPGASA
AAAAAGGYGPGSGQQGPGQQGPGQQGPGGQ*ASASASAAASAASTVANSSS*NLK
VEFYNSNPSDTTNSINPQFKVTNTGSSAIDLSKLTLRYYYTVDGQKDQTFWC
DHAAIIGSNGSYNGITSNVKGTFVKMSSSTNNADTYLEISFTGGTLEPGAHVQ
IQGRFAKNDWSNYTQSNDYSFKSASQFVEWDQVTAYLNGVLVWGKE*LEHH*
HHHH

FN-ADF3-FN sequence

Structure: Terminal domain (FN)-*Linker*-Mid-block (ADF3)-*Linker*-Terminal domain (FN)-*His-tag*

Sequence:

VSDVPRDLEVVAATPTSLLISWDAPAVTVRYYRITYGETGGNSPVQEFTVPG
SKSTATISGLKPGVDYTITVYAVTGRGDSPASSKPISINYRTEI*PSASASASAGAS*
*AAASAGAGAGA*GPGQQGPGQQGPGQQGPYGPGASAAAAAAGGYGPGSGQQ
GPSQQGPGQQGPGGQGPYGPGASAAAAAAGGYGPGSGQQGPGGQGPYGPG
SSAAAAAAGGNGPGSGQQGAGQQGPGQQGPGGSAAAAAAGGYGPGSGQQ

GPGQQGPGGQGPYGPGASAAAAAAGGYGPGSGQGPGQQGPGGQGPYGPG
ASAAAAAAGGYGPGSGQQGPGQQGPGQQGPGGQGPYGPGASAAAAAAGG
YGPGYGQQGPGQQGPGGQGPYGPGASAASAASGGYGPGSGQQGPGQQGPG
GQGPYGPGASAAAAAAGGYGPGSGQQGPGQQGPGQQGPGQQGPGGQGPY
GPGASAAAAAAGGYGPGSGQQGPGQQGPGQQGPGQQGPGQQGPGQQGPG
QQGPGQQGPGQQGPGGQGAYGPGASAAAGAAGGYGPGSGQQGPGQQGPG
QQGPGQQGPGQQGPGQQGPGQQGPGQQGPYGPGASAAAAAAGGYGPGSG
QQGPGQQGPGQQGPGGQ*ASASASAAASAASTVANSSS*VSDVPRDLEVVAATPTS
LLISWDAPAVTVRYYRITYGETGGNSPVQEFTVPGSKSTATISGLKPGVDYTI
TVYAVTGRGDSPASSKPISINYRTEI*LEHHHHHH*

SpyC2-ADF3-SpyC2 sequence

Structure: Terminal domain (SpyC2)-*Linker*-Mid-block (ADF3)-*Linker*-Terminal domain (SpyC2)-*His-tag*

MGAMVTTLSGLSGEQGPSGDMTTEEDSATHIKFSKRDEDGRELAGATMELR
DSSGKTISTWISDGHVKDFYLYPGKYTFVETAAPDGYEVATAITFTVNEQGQ
VTVNGEATKGDAHT*SASASASAGASAAASAGAGAGA*GPGQQGPGQQGPGQQG
PYGPGASAAAAAAGGYGPGSGQQGPSQQGPGQQGPGGQGPYGPGASAAAA
AAGGYGPGSGQQGPGGQGPYGPGSSAAAAAAGGNGPGSGQQGAGQQGPG
QQGPGGSAAAAAAGGYGPGSGQQGPGQQGPGGQGPYGPGASAAAAAAGG
YGPGSGQGPGQQGPGGQGPYGPGASAAAAAAGGYGPGSGQQGPGQQGPG
QQGPGGQGPYGPGASAAAAAAGGYGPGYGQQGPGQQGPGGQGPYGPGAS
AASAASGGYGPGSGQQGPGQQGPGGQGPYGPGASAAAAAAGGYGPGSGQ
QGPGQQGPGQQGPGQQGPGGQGPYGPGASAAAAAAGGYGPGSGQQGPGQ

QGPGQQGPGQQGPGQQGPGQQGPGQQGPGQQGPGQQGPGGQGAYGPGAS
AAAGAAGGYGPGSGQQGPGQQGPGQQGPGQQGPGQQGPGQQGPGQQGPG
QQGPYGPGASAAAAAAGGYGPGSGQQGPGQQGPGQQGPGGQ*ASASASAAAS*
*AASTVANSSS*AMVTTLSGLSGEQGPSGDMTTEEDSATHIKFSKRDEDGRELAG
ATMELRDSSGKTISTWISDGHVKDFYLYPGKYTFVETAAPDGYEVATAITFT
VNEQGQVTVNGEATKGDAHTS*LEHHHHHH*

Crys-AQ12-Crys sequence

Structure: Terminal domain (Crys)-*Linker*-Mid-block (AQ12)-*Linker*-Terminal domain (Crys)-*His-tag*

MGKITLYEDRGFQGRHYECSSDHPNLQPYLSRCNSARVDSGCWMLYEQPNY
SGLQYFLRRGDYADHQQWMGLSDSVRSCRLIPHSGSHRIRLYEREDYRGQM
IEFTEDCSCLQDRFRFNEIHSLNVLEGSWVLYELSNYRGRQYLLMPGDYRRY
QDWGATNARVGSLRRVIDFS*PSASASASAGASAAASAGAGAG*AGPYGPGASAA
AAAAGGYGPGSGQQGPGQQGPGQQGPGQQGPGQQGPYGPGASAAAAAAG
GYGPGSGQQGPGQQGPGQQGPGQQGPGQQGPYGPGASAAAAAAGGYGPG
SGQQGPGQQGPGQQGPGQQGPGQQGPYGPGASAAAAAAGGYGPGSGQQG
PGQQGPGQQGPGQQGPGQQGPYGPGASAAAAAAGGYGPGSGQQGPGQQG
PGQQGPGQQGPGQQGPYGPGASAAAAAAGGYGPGSGQQGPGQQGPGQQG
PGQQGPGQQGPYGPGASAAAAAAGGYGPGSGQQGPGQQGPGQQGPGQQG
PGQQGPYGPGASAAAAAAGGYGPGSGQQGPGQQGPGQQGPGQQGPGQQG
PYGPGASAAAAAAGGYGPGSGQQGPGQQGPGQQGPGQQGPGQQGPYGPG
ASAAAAAAGGYGPGSGQQGPGQQGPGQQGPGQQGPGQQGPYGPGASAAA
AAAGGYGPGSGQQGPGQQGPGQQGPGQQGPGQQGPYGPGASAAAAAAGG

YGPGSGQQGPGQQGPGQQGPGQQGPGQQ*ASASASAAASAASTVANSSS*GKITL

YEDRGFQGRHYECSSDHPNLQPYLSRCNSARVDSGCWMLYEQPNYSGLQYF

LRRGDYADHQQWMGLSDSVRSCRLIPHSGSHRIRLYEREDYRGQMIEFTEDC

SCLQDRFRFNEIHSLNVLEGSWVLYELSNYRGRQYLLMPGDYRRYQDWG

TNARVGSLRRVIDFS*LEHHHHHH*

Stability of the hydrophobic silk film when measuring static contact angle

Static CA measurement over a longer period of time was performed to study the stability of the silk films. Silk films from 0.3 mg/ml of Crys-ADF3-Crys (in 0.5 mM Tris-HCl) were prepared on a glass slide and dried overnight at 80% RH as described in the Materials and Methods section. The static CA of a silk film was measured as described in the Materials and Methods section, with the exception of increasing the acquisition time to 30 minutes and recording at 0.033 frames per second (**Figure S1**). Also, to minimize evaporation of the water droplet, the film was covered with a transparent shield. During the 30-minute measurement period, only a minor decrease in CA was observed from 126° to 123°. This steady decrease in CA was caused by the slow evaporation of the water droplet. The baseline between the water droplet and the silk film remained constant at 1.77 mm, and thus the shrinking of the water droplet caused the contact angle to decrease. Spreading of the water droplet on the silk film was not observed.

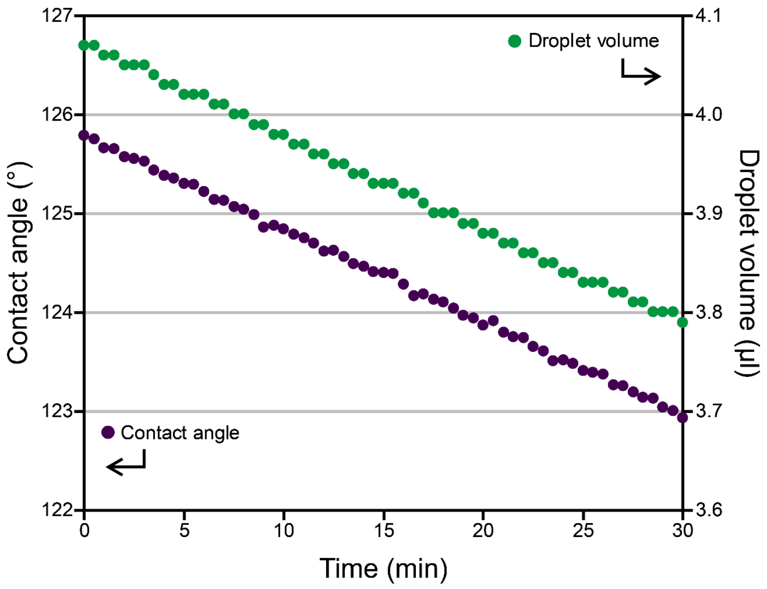

Figure S1. *Static contact angle of a hydrophobic silk film over a longer period of time. The silk film was prepared from 0.3 mg/ml of Crys-ADF3-Crys and dried overnight at 80% RH. A small change in contact angle was observed during the 30-minute period due to the slow evaporation of the water droplet. No spreading of the droplet on the silk film was observed.*

Static contact angle of silk films on different support materials

Silk films were prepared on top of different flat supporting materials to test whether the hydrophobicity is affected by the underlying support material. Films were prepared by pipetting 50 µl of 0.3 mg/ml Crys-ADF3-Crys (in 0.5 mM Tris-HCl pH 7.4) and dried overnight at 80% RH, as described in the Materials and Methods section. Five different support materials were tested; three different glass slides that varied in their hydrophobicity (static water CA ~55°, ~64° and ~100°), polystyrene slide (static CA ~89°) and smooth aluminium sheet. CA of the aluminium sheet was not measured.

The static CAs of the silk films were measured as described in the Materials and Methods section (**Figure S2**). One-way analysis of variance was performed between the sample groups and no correlation was found between the CA value and different support materials (p-value 0.464), indicating that the support material does not have a significant effect on the hydrophobicity of the silk films. However, very hydrophilic support materials, such as mica, proved to be difficult to coat with the same method as the 50 µl volume of silk solution spread over a very large area. This led to much thinner and uneven coatings.

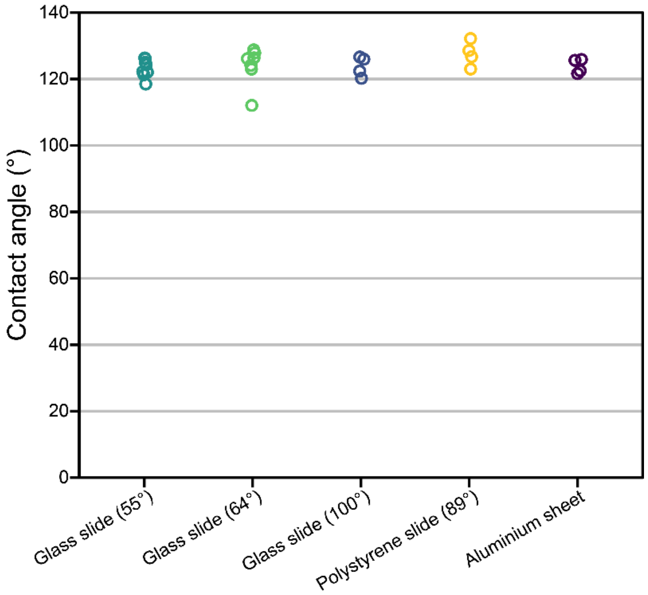

Figure S2. *Contact angle of silk films coated on different surfaces. The silk films were prepared from 0.3 mg/ml of Crys-ADF3-Crys and dried overnight at 80% RH. The results show no significant difference between different support materials (p-value 0.464).*

Zisman plot and oil wetting of silk films

The wetting of the hydrophobic silk film by isopropanol (IPA) - water mixtures (0-32 vol% IPA) and oils (hexadecane and silicon oil) were measured to determine the critical surface tension of the hydrophobic silk film. First, surface tension (ST) of IPA-water mixtures and oils were measured with a pendant droplet method using an optical tensiometer (Theta Flex, Biolin Scientific). The liquid of interest was dispensed from a pipet tip until the downwards hanging droplet had a pendant like shape. The measurement was recorded for 15 seconds at 14 frames per second. The ST value was taken from an average of 3 repetitions. ST of isopropanol-water mixtures are shown in **Table S1**. ST of silicon oil (10 cst) and hexadecane were 19.6 and 27.1 mN/m, respectively.

Table S1. Surface tension of isopropanol-water dilution series.

Isopropanol in water (vol%)	Surface tension (mN/m)
0	71.5
1	62.2
2	55.6
4	49.6
8	45.8
16	33.5
24	26.4
32	22.2

Next, the static CA (θ) of each liquid was measured as described in the Materials and Methods section, which were then plotted in format of 1-cos(θ) against the ST of the liquid (**Figure S3**). The critical surface tension of the film was determined to be ~22

mN/m when using the IPA-water mixtures. However, also hexadecane (ST of 27.1 mN/m) did fully wet the films.

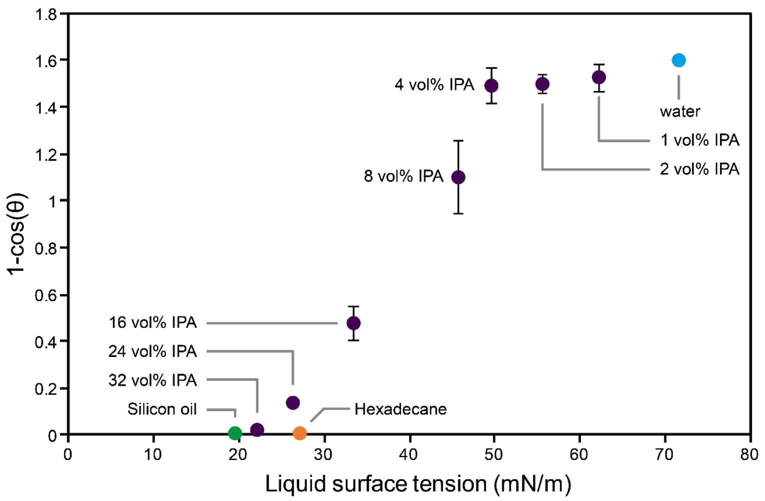

Figure S3*. Zisman plot of hydrophobic silk film. Static contact angle (θ) of the silk film was measured with isopropanol (IPA) - water dilution series (1-32 vol% IPA in water), water, silicon oil, and hexadecane, which were plotted against the surface tension of the liquids. Critical surface tension of the silk film was ~22 mN/m when using IPA-water mixture. However, also hexadecane with surface tension of 27.1 mN/m did fully wet the silk films. The silk films were prepared from 0.3 mg/ml of Crys-ADF3-Crys in 0.5 mM Tris-HCl and dried overnight at 80% RH. Error bars are ±SD (3 measurements per liquid).*

X-ray photoelectron spectroscopy of silk films prepared at 35 and 80% RH

The hydrophobicity of the silk films prepared at high humidity could originate from the silk protein folding in a way that the hydrophobic alanine blocks stack on the surface. The alanine blocks of the Crys-ADF3-Crys are located in the ADF3 section. Consequently, the Crys terminal domains would be further away from the surface. The

chemical bonding and composition of the surface layer of the sample can be analysed with X-ray photoelectron spectroscopy (XPS). The likelihood of photoelectron emission decreases rapidly with increasing depth in the sample, which makes XPS a very surface sensitive technique, with a total maximum probing depth of around 5-10 nm. The peptide bond itself contains C-C, C-O, C=O, C-N, and -N-C=O bonds, which complicates identifying many of the amino acids. However, crystallin is rich in carboxyl (O-C=O) and hydroxyl (C-O) containing amino acids, 13% and 34% of total amino acids in crystallin, respectively, in comparison to 0 and 10% in ADF3. Thus, differences in C-O and O-C=O bonding environments could indicate which part of the protein is closer to the surface as illustrated in **Figure S4**. The results show clear differences in the molecular composition and bonding in the silk films between the two preparation methods (**Table S2-5**, **Figure S5-8**). The 80% RH films contain relatively more chloride salts and nitrogen, as well as C-N, C=O, and -NH bonding. The 35% RH films contain more carbon and oxygen, as well as C-C, C-O, O-C=O, -N-C=O, -NOH, and C-OH bonding environments. From these results, it is evident that the surface of the film is different between the two preparation methods. The lower content of C-O and O-C=O environments in 80% RH films suggests higher content of ADF3 on the surface in comparison to 35% RH films, which supports the hypothesis that the hydrophobicity originates from the alanine blocks of the ADF3. Further details of the results are discussed below.

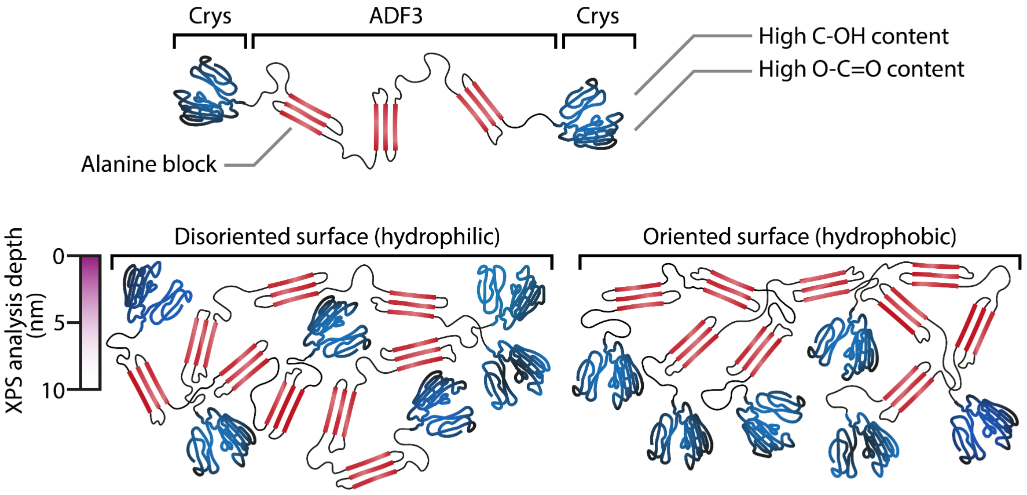

Figure S4. *Illustration of the silk proteins on the film surface. Not drawn in accurate scale.*

The relative composition of the samples can be seen in **Table S2**. Minor differences in the elemental compositions were detected for the different films, while films of the same type gave very similar results. All values in **Table S2-5** are averages from measurements on two different positions on each measured film, where a and b denote separate films of each type. Survey spectra is shown in **Figure S5**.

Table S2. *Relative concentrations of elements in all samples (35 vs 80% RH films). The two final columns give the O/C and N/C ratio for all films.*

Sample	C 1s %	Cl 2p %	N 1s %	Na 1s %	O 1s %	O/C	N/C
1 80% RH a	63.54	3.50	15.02	0.31	17.63	0.28	0.00
1 80% RH b	61.71	3.76	15.65	0.33	18.56	0.30	0.25
2 35% RH a	66.47	0.34	13.49	0.00	19.70	0.30	0.20
2 35% RH b	65.80	1.35	13.07	0.05	19.74	0.30	0.20

Table S3 shows the relative ratios of the different components of carbon in samples. The C 1s spectra (**Figure S6**) were fitted with five Gaussian components according to standard tabulated chemical shifts, with peak positions at 284.8 eV (C-C), 285.6 eV (C-N), 286.5 eV (C-O), 287.8 eV (C=O), and 288.9 eV (O-C=O). Very similar shapes were measured for the spectra from the two different films of either type, while significant difference could be seen between the different types. The small energy difference, especially between the C-N and C-O components, slightly affects the credibility of the ratios between the different components, causing the ratio of carbon components to be slightly different than what the elemental composition would suggest.

Table S3*. Relative amounts of the different components of carbon, as compared to the total amount of carbon in the samples (35 vs 80% RH films).*

Sample	C (C-C) %	C (C-N) %	C (C-O) %	C (C=O) %	C (O-C=O) %
1 80% RH a	32.74	23.12	20.87	22.37	0.89
1 80% RH b	32.79	26.57	17.60	22.01	1.03
2 35% RH a	39.91	16.28	23.04	19.19	1.58
2 35% RH b	38.81	16.23	25.30	18.37	1.29

Three components of nitrogen could be observed in the N 1s region (**Figure S7**), located at approximately 399.9 eV, 401.3 eV, and 402.3 eV. Most likely these correspond to nitrogen in amides (-N-C=O), amines (-NH), and hydroxylamines (-NOH), respectively. A clear and consistent difference can be seen for the different types of films. **Table S4** gives the relative concentrations of each component of nitrogen in the samples.

Table S4*. Relative amounts of the different components of nitrogen, as compared to the total amount of nitrogen in the samples (35 vs 80% RH films).*

Sample	N (-N-C=O) %	N (-NH) %	N (-NOH) %
1 80% RH a	83.98	13.18	2.84
1 80% RH b	85.19	14.11	0.71
2 35% RH a	96.48	2.45	1.07
2 35% RH b	91.89	6.49	1.62

Table S5 shows the relative amounts of oxygen in the different samples. The O 1s spectra (**Figure S8**) were fitted with two Gaussian components at approximately 531.5 eV and 532.7 eV, these correspond to oxygen and carbon double bonds and single bonds, respectively. Here again, the relative concentrations are clearly different between the two film types, and also consistent between the two films of each type, although differences are very minor (as perhaps can be expected).

Table S5*. Relative amounts of the different components of oxygen, as compared to the total amount of oxygen in the samples (35 vs 80% RH films).*

Sample	O (C-OH) %	O (C=O) %
1 80% RH a	36.00	64.00
1 80% RH b	37.45	62.55
2 35% RH a	41.72	58.28
2 35% RH b	45.45	54.55

In addition to the previously mentioned regions, the Cl 2p region again exhibited a typical doublet with the 2p3/2 peak located approximately 197.8 eV, which is typical for a chloride. All chloride was found to be in this form.

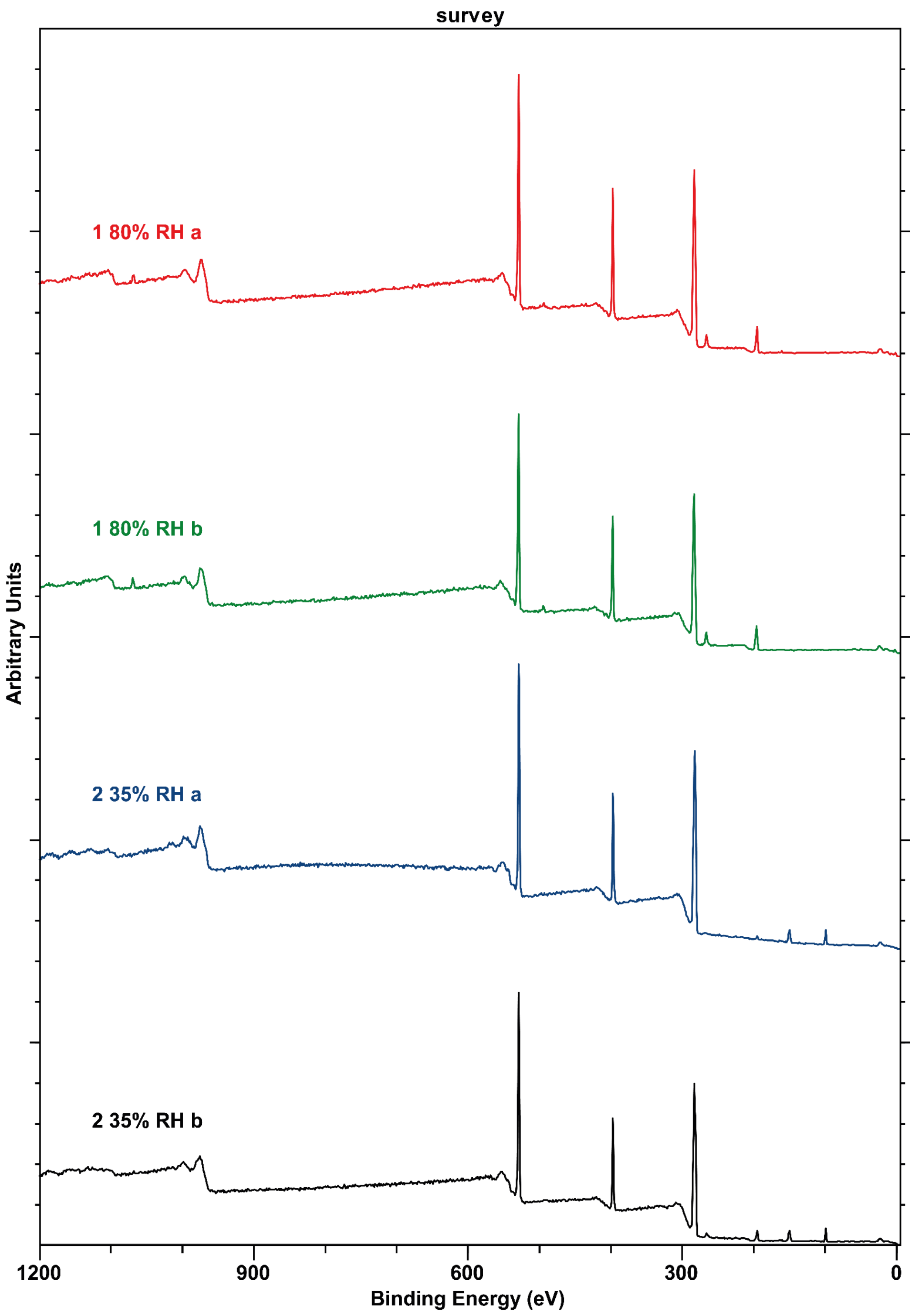

Figure S5. *XPS survey spectra of silk films prepared at 35 and 80% RH (two replicates).*

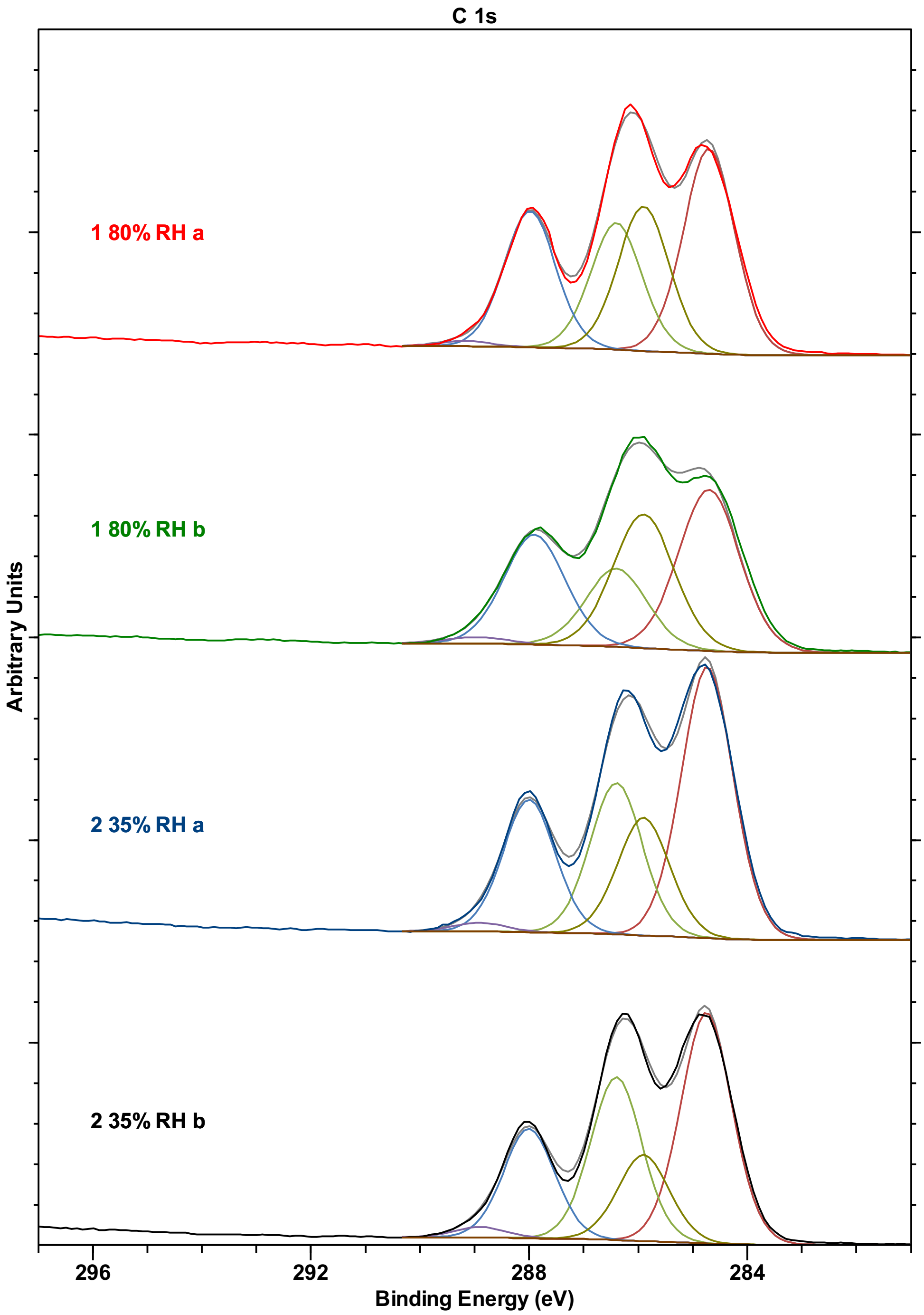

Figure S6. *XPS high-resolution C 1s spectra of silk films prepared at 35 and 80% RH (two replicates).*

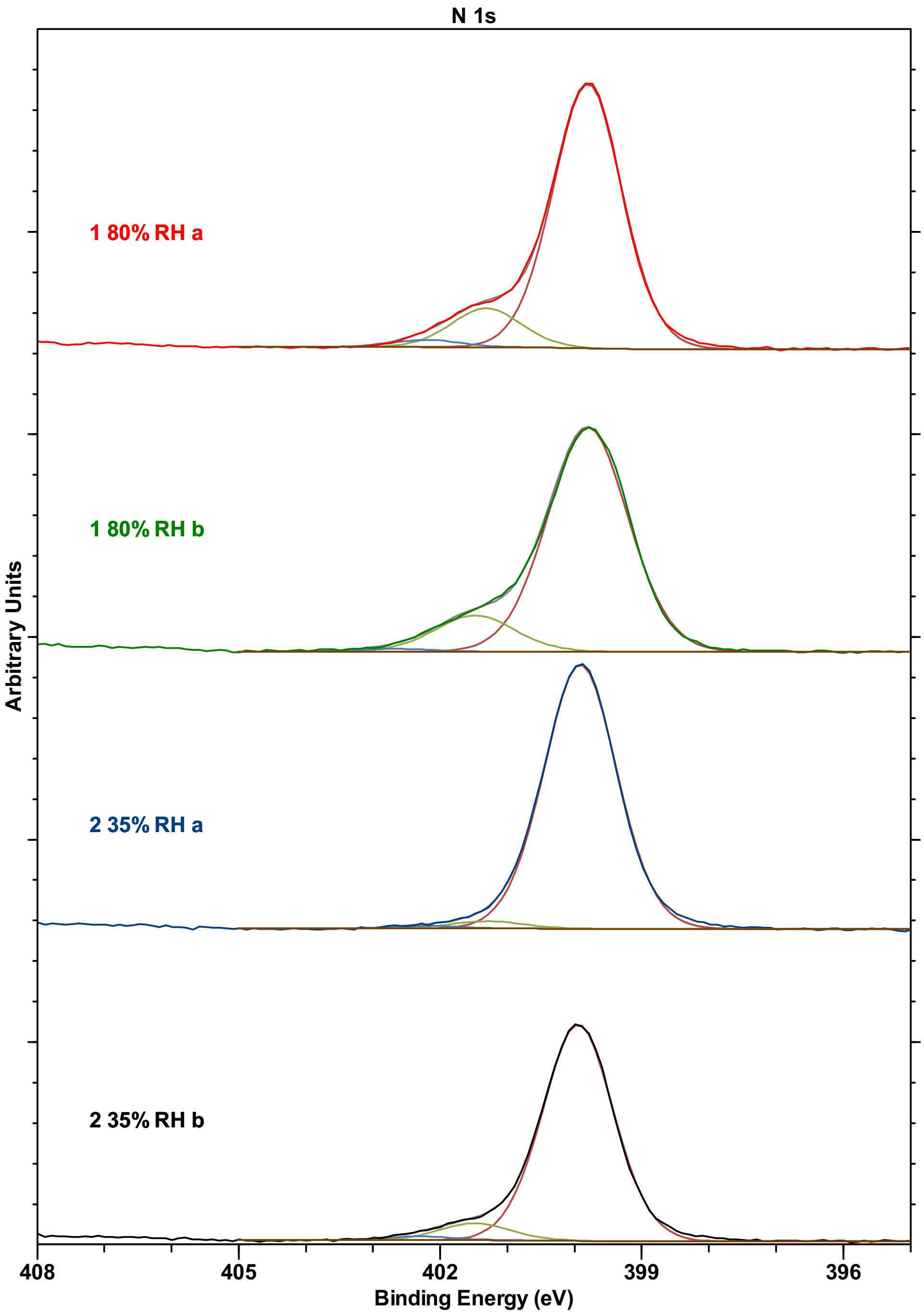

Figure S7. *XPS high-resolution N 1s spectra of silk films prepared at 35 and 80% RH (two replicates).*

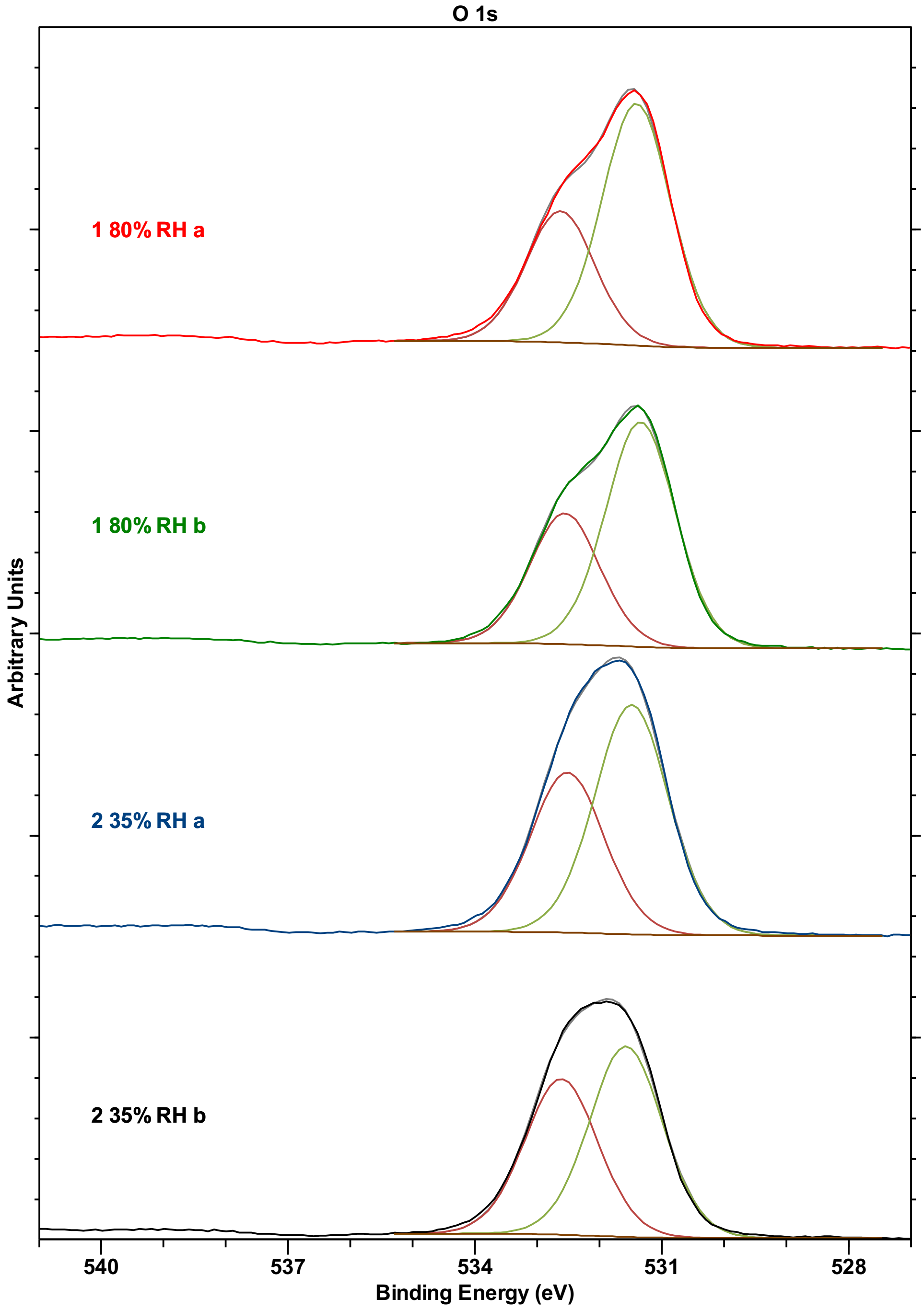

Figure S8. *XPS high-resolution O 1s spectra of silk films prepared at 35 and 80% RH (two replicates).*

AFM of silk films prepared at 80% RH

Topographical characterization of silk films prepared at 80% RH, considering an area of 10x10 µm. The scan speed was 79.37 µm/s with a resolution of 128 samples per line resolution in quantitative imaging QI mode. Different areas were characterized. The height of scanned areas shows that the film did reach up to 532 nm due to the presence of aggregates. The average Ra roughness was 19.64 nm ±9.85 nm, and RMS roughness was 26.4 nm ±13.1 nm.

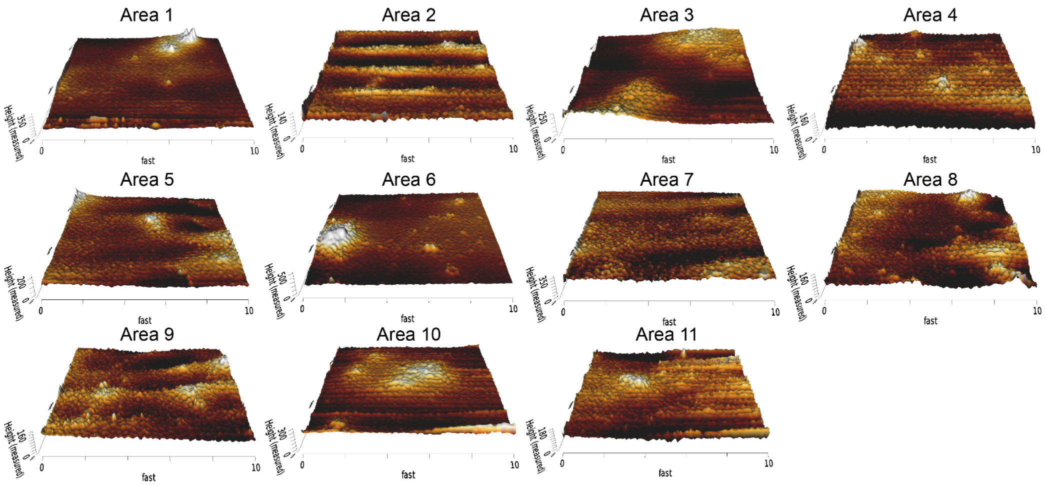

Figure S9. *AFM - Topography of silk films prepared at 80% RH. 11 different 10x10 µm areas were measured. X-axis unit is µm, y-axis unit is nm.*

AFM of silk films prepared at 35% RH

Topographical characterization of silk films prepared at 35% RH, considering an area of 10x10 µm. The scan speed was 79.37 µm/s with a resolution of 128 samples per line resolution in quantitative imaging QI mode. The measured height did reach up to 500 nm if the area contained aggregates. Nevertheless, the average Ra roughness of 35% RH was 9.28 nm ±1.85 nm, and average RMS roughness was 12.5 nm ±2.6 nm.

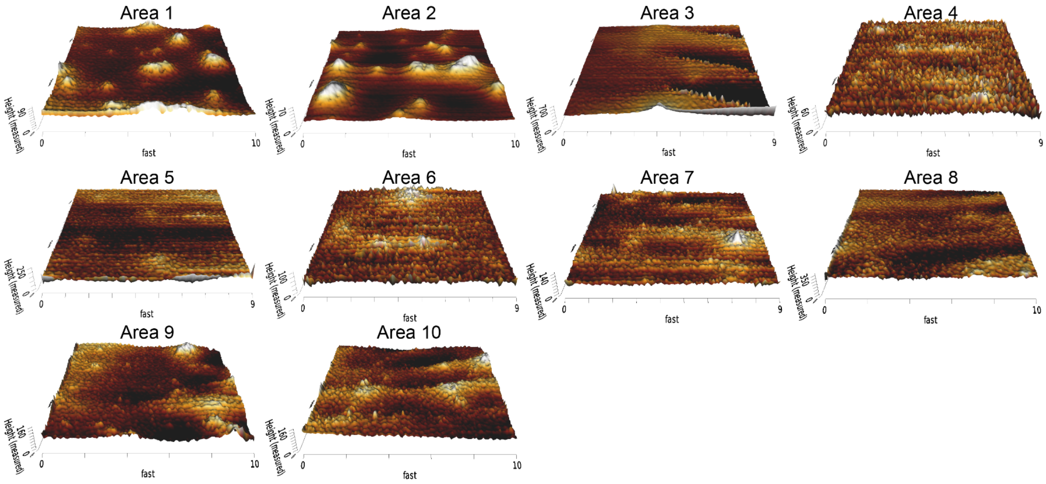

Figure S10. *AFM – Topography of silk films prepared at 35% RH. 10 different 9-10x9-10 µm areas were measured. X-axis unit is µm, y-axis unit is nm.*

AFM – Young's modulus of the silk films

Values of young's modulus of the silk films (**Figure S11**) were obtained together with the AFM topographical characterization. The data points were combined from 11 and 10 different areas of silk films prepared at 35 and 80% RH, respectively. Spread of the data points differ between the two preparation methods: Young's modulus of 35% RH films are more clustered at ~42 kPa while 80% RH films have two clusters at 73 kPa and 13 kPa, suggesting that 80% RH films have slightly softer and harder regions. However, no significant difference was found between the median values of the individual sample areas (Wilcox-test p-value 0.999).

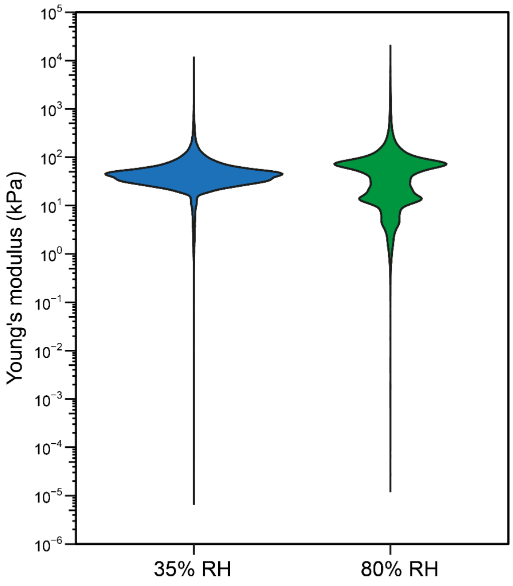

Figure S11. *AFM – Young's modulus of silk films prepared at 35 and 80% RH. Data points of 35 and 80% RH are combined from 10 and 11 areas, respectively. Median values of 35 and 80% RH are 41.7 and 42.0 kPa, respectively.*

Nanosized filaments in silk films prepared at 35% RH

Films of Crys-ADF3-Crys were prepared at 35% RH on rough aluminium SEM stubs, which caused cracks on the film during the drying step. To study the internal structure of the silk films, the samples were observed under SEM, and films showed nanosized silk filaments on the edges of the cracks (**Figure S12a**, **Figure S12b**) and unusually rough film surface (**Figure S12c**).

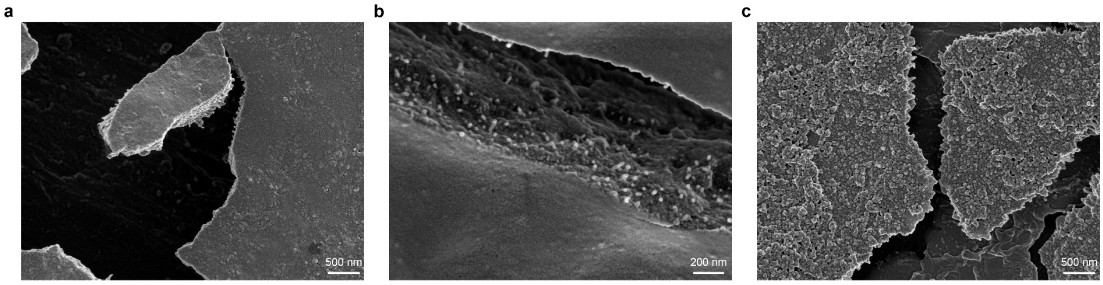

Figure S12. *Cracked silk films on aluminium stub, prepared at 35% RH.*

Crystallin and BSA films

BSA and crystallin films were prepared with the same method as with silk films, which is described in the Materials and Methods section. Both films were hydrophilic (**Figure S13**). Crystallin was used to demonstrate that the terminal domain crystallin itself is not the main cause of the hydrophobicity of the silk films. BSA was used to show that the hydrophobicity of silk films is not inherent for protein films. 0.3 mg/ml of protein was used in 0, 0.5 and 1 mM of Tris-HCl (pH 7.4) and the incubation was done at 80% RH. The static CAs of the silk films were measured as described in the Materials and Methods section.

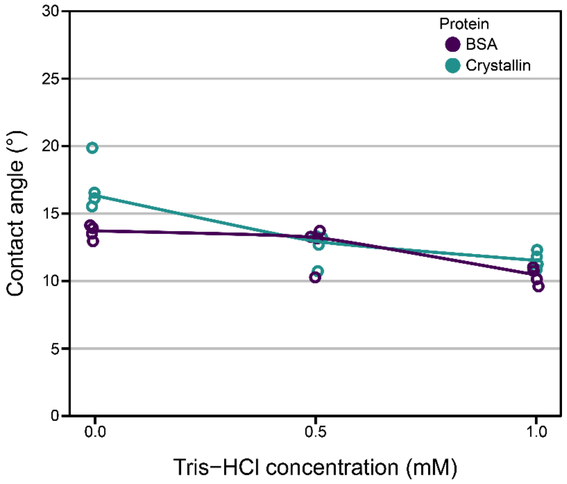

Figure S13. *Contact angle of crystallin and BSA films (0.3 mg/ml of protein in 0-1.0 mM of Tris-HCl) prepared at 80% RH.*

Crystal-like structures after wetting of the silk film

The silk films showed structural changes after being wetted by water. Silk films were prepared from 0.3 mg/ml of Crys-ADF3-Crys in 0.5 mM Tris-HCl, and incubated at 80% RH, as described in materials and methods section. A droplet of distilled water

(4 μl) was placed on the films and dried again overnight at 80% RH (20°C). The films were coated with 5 nm of Au/Pd and imaged with SEM. The images show structural changes under the area that was wetted. Crystal-like structures had appeared that were not visible on non-wetted surfaces (**Figure S14**, **Figure S15**). These structures sometimes formed curved patterns as shown in **Figure S15b**.

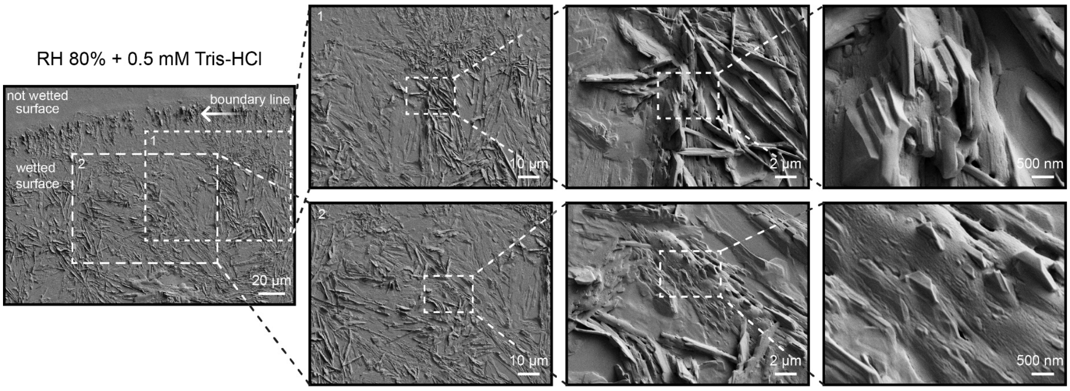

Figure S14. *Crystal-like structures in a silk film after wetting by water and drying.*

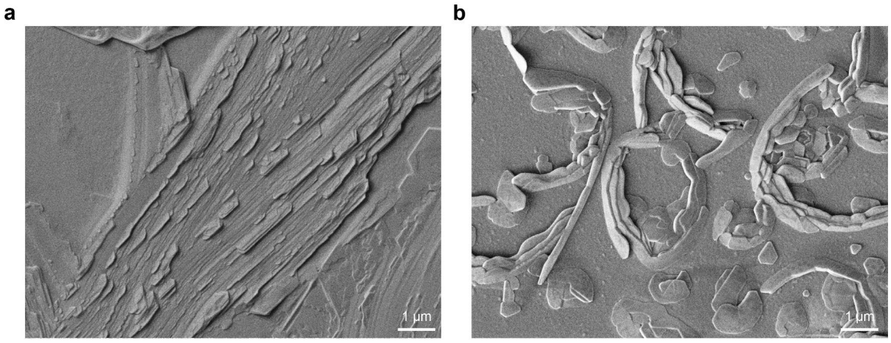

Figure S15. *Crystal-like structures in different patterns in silk films after wetting by water and drying. (a) Crystal-like structures in straight patterns. (b) Crystal-like structures in curved patterns.*

Contact angle before and after wetting of the silk film

The silk films showed structural changes after being wetted by water. Silk films were prepared from 0.3 mg/ml of Crys-ADF3-Crys in 0.5 mM Tris-HCl, and incubated at 80% RH, as described in the Materials and methods section. First, CAs of the films were measured ("Before" in **Figure S16**), and then the films were then submerged under ~100 µl of distilled water for 5 minutes. The water was aspirated, and the films were dried overnight at 80% RH (20°C) and the CA of the films were measured again ("After" in **Figure S16**). CA of the naked glass was 55°.

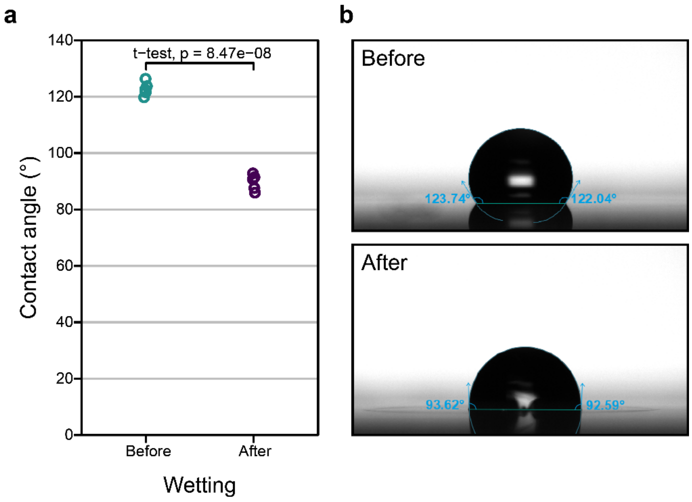

Figure S16. *Changes in contact angle (CA) of the silk films after wetting by water: the CA of freshly prepared silk film was measured ("Before"), the silk film was submerged in water and dried, and the CA was measured again ("After").*

X-ray photoelectron spectroscopy of the wetted and non-wetted area of the high CA silk film

The wetted high CA silk films were analysed with XPS to determine the compositional differences between regions of the silk film that had been wetted by a droplet of water and the original dry film. The wetted films were dried again overnight at 80% RH (20°C). XPS is a very surface sensitive technique. Results show that the wetted regions of the silk surface have increased concentrations of chlorine, together with higher levels of C-O bonded carbon and nitrogen in the form of amines (**Table S6-10, Figure S17-22**). This would indicate that any crystalline structures in these regions could be caused by increased amounts of Tris-HCl residue. Further details of the results are discussed below.

The relative composition of the samples can be seen in **Table S6**. The main difference between the dry and wetted areas is an increase in the wetted areas of the concentrations of chlorine, oxygen, and sodium, at the expense of a decrease in the amount of carbon. As a reference, the composition of the glass slide was also determined to consist of mainly silicon and oxygen, with small amounts of potassium and sodium as well as carbon contamination. Survey spectra (**Figure S17**) also showed minor traces of boron and zinc on the uncoated glass slide.

Table S6*. Relative concentrations of elements in all samples (wetted and dry film).*

Sample	C 1s %	Cl 2p %	K 2p %	N 1s %	Na 1s %	O 1s %	Si 2p %
Silk wetted	51.30	6.68	0.38	16.39	0.85	24.41	0.00
Silk dry	62.44	2.22	0.00	15.89	0.10	19.35	0.00
Glass slide	15.13	0.00	3.24	0.00	2.27	52.52	26.84

All values in **Table S6-10** are averages from all three positions on the different samples (with the exception of the values for the wetted silk, calculated for only two points, as the third point was omitted due to severe charging effects).

Table S7 shows the relative ratios of the different components of carbon in samples. The C 1s spectra (**Figure S18**) were fitted with five Gaussian components according to standard tabulated chemical shifts, with peak positions at 284.8 eV (C-C), 285.6 eV (C-N), 286.5 eV (C-O), 287.8 eV (C=O), and 288.9 eV (O-C=O). The small energy difference between the C-C, C-N, and C-O components affects the credibility of the ratios between the different components, where ideally the amount of C-N carbon should be much higher for pure silk. A shift toward higher energies, and larger amounts of C-O, for the wetted part of the film does, however, hint toward a higher concentration of C-O bonding in these regions.

Table S7*. Relative amounts of the different components of carbon, as compared to the total amount of carbon in the samples (wetted and dry film).*

Sample	C (C-C) %	C (C-N) %	C (C-O) %	C (C=O) %	C (O-C=O) %
Silk wetted	29.84	6.24	41.59	17.83	4.50
Silk dry	27.93	15.25	33.06	19.38	4.37
glass slide	3.91	0.00	72.69	20.87	2.53

Three components of nitrogen could be observed in the N 1s region (**Figure S19**), located at approximately 399.9 eV, 401.3 eV, and 402.3 eV. Most likely these correspond to nitrogen in amides (-N-C=O), amines (-NH), and hydroxylamines (-NOH), respectively. A clear increase in the levels of amines and hydroxylamines can be seen for the wetted regions of the silk film. **Table S8** gives the relative concentrations of each component of nitrogen in the samples.

Table S8. *Relative amounts of the different components of nitrogen, as compared to the total amount of nitrogen in the samples (wetted and dry film).*

Sample	N (-N-C=O) %	N (-NH) %	N (-NOH) %
Silk wetted	71.34	20.16	8.50
Silk dry	86.97	9.69	3.34
glass slide	0.00	0.00	0.00

Table S9 shows the relative amounts of oxygen in the different samples. The O 1s spectra (**Figure S20**) for the wetted and dry silk were fitted with two Gaussian components at approximately 531.7 eV and 532.9 eV, these correspond to oxygen and carbon double bonds and single bonds, respectively. For the dry silk most oxygen is bound with double bonds, while the amount of oxygen in single bonds increases for the wetted region. In this respect the O 1s gives a different picture than the C 1s spectra, but this is most likely just a reflection of the inaccuracies of the C 1s fitting. Ratios giving here are likely more accurate, as C-N bonding does not affect the O 1s spectra. The glass slide exhibits higher energy bonding, with an oxide peak present at approximately 533.0 eV, typical for SiO_2, and a peak at 534.5 eV, most likely corresponding to surface hydroxyls.

Table S9. *Relative amounts of the different components of oxygen, as compared to the total amount of oxygen in the samples (wetted and dry film).*

Sample	O (C-OH) %	O (C=O) %	O (OH) %	O (oxide) %
Silk wetted	48.99	51.01	0.00	0.00
Silk dry	33.31	66.69	0.00	0.00
glass slide	0.00	0.00	67.81	32.19

Sodium levels were fairly low in all regions of the sample, but three distinct peaks could nonetheless be observed in the Na 1s region (**Figure S21**). The lower energy peak, at 1071.5 eV, is a typical energy for most sodium in the form of Na (I). The higher energy components, at 1074.1 eV and 1075.9 eV, cannot easily be found in literature references, as sodium seldom appears at energies above 1073 eV. These higher energy peaks can very likely be related to a sodium oxide (Na_2O), but they might also be caused by an interaction between photoelectrons from the main Na (I) peak and carbon at the surface layers of the sample. High binding energy electrons have very low kinetic energy, and therefore have a higher likelihood of interaction with the electrons from other elements in the samples. Carbon materials are notorious for their adverse effect on the intensities of photoelectrons from, among others, sodium. **Table S10** shows the relative amounts of all sodium components in the different samples, with the higher binding energies most prevalent on the area of the uncoated glass slide.

In addition to the previously mentioned regions, the Cl 2p region (**Figure S22**) exhibited a typical doublet with the $2p_{3/2}$ peak located approximately 197.8 eV, which is typical for a chloride. All chlorine was found to be in this form. All silicon on the glass slide was also found to be bound in SiO_2, with a typical binding energy of about 103.6 eV.

Table S10*. Relative amounts of the different components of sodium, as compared to the total amount of sodium in the samples (wetted and dry film).*

Sample	Na (1071.5) %	Na (1074.1) %	Na (1075.9) %
Silk wetted	56.96	15.42	27.63
Silk dry	94.86	4.48	0.65
glass slide	10.16	19.71	70.13

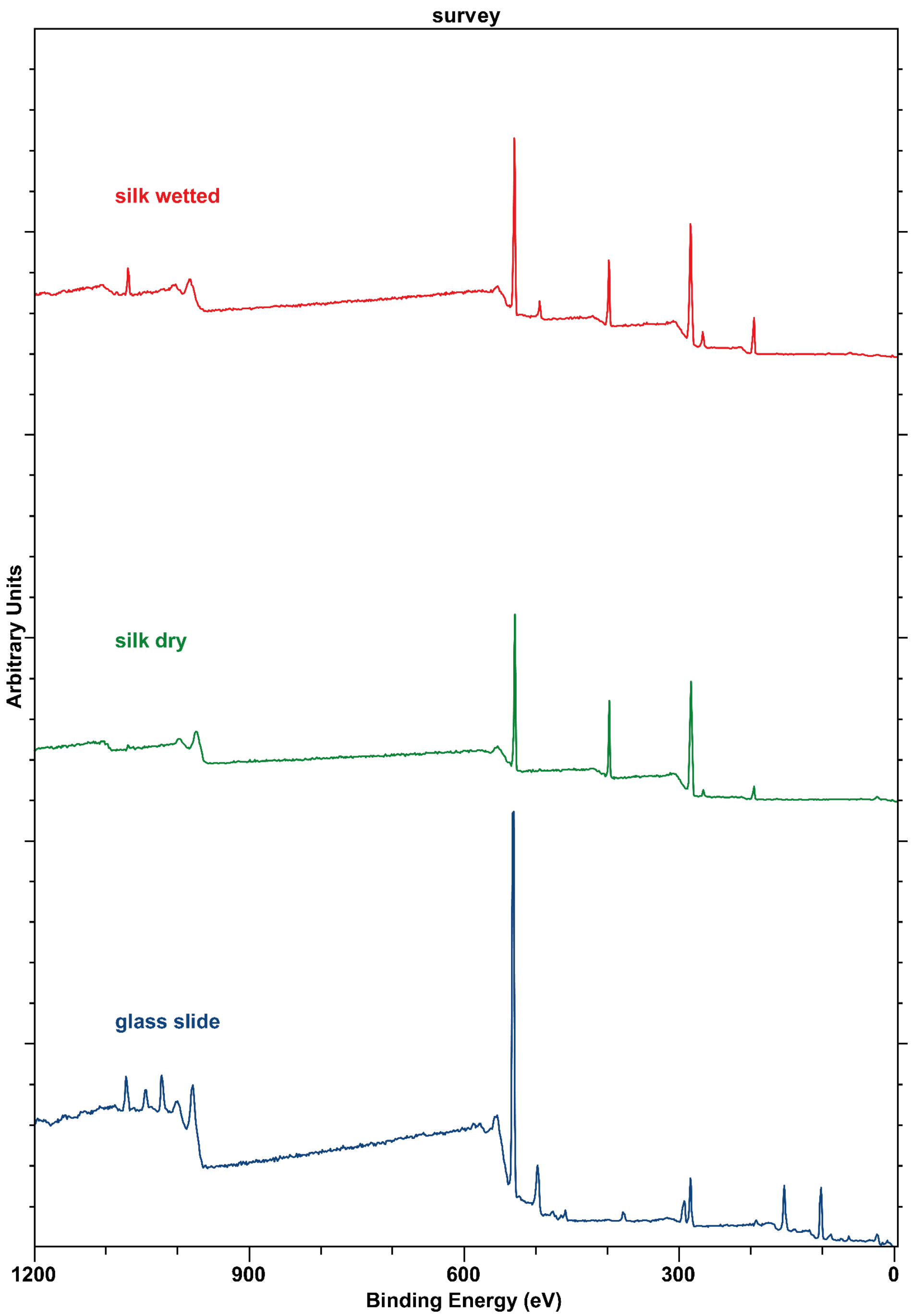

Figure S17. *XPS survey spectra of high CA silk films (wetted and dry) and a glass slide.*

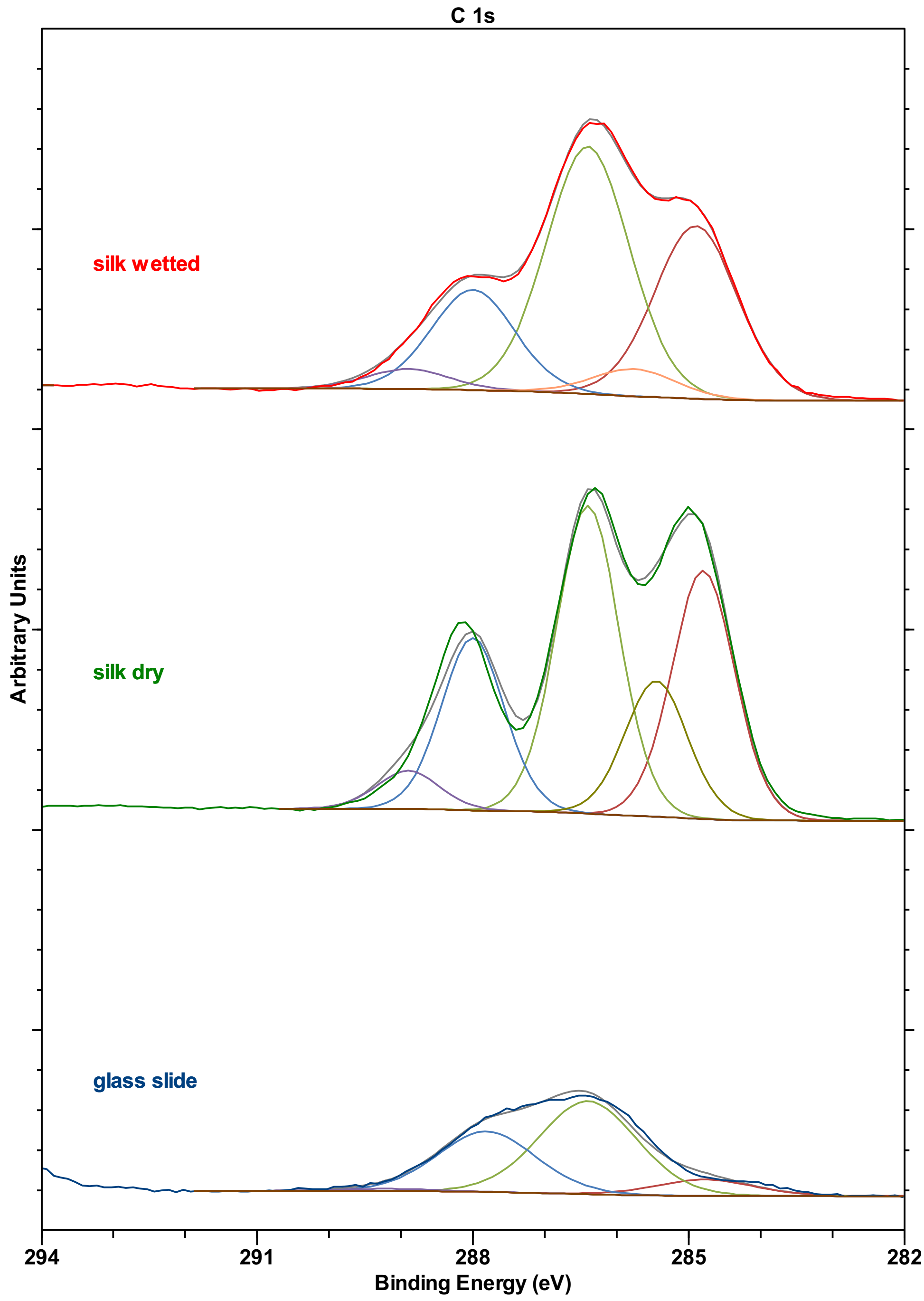

Figure S18. *XPS high-resolution C 1s spectra of high CA silk films (wetted and dry) and a glass slide.*

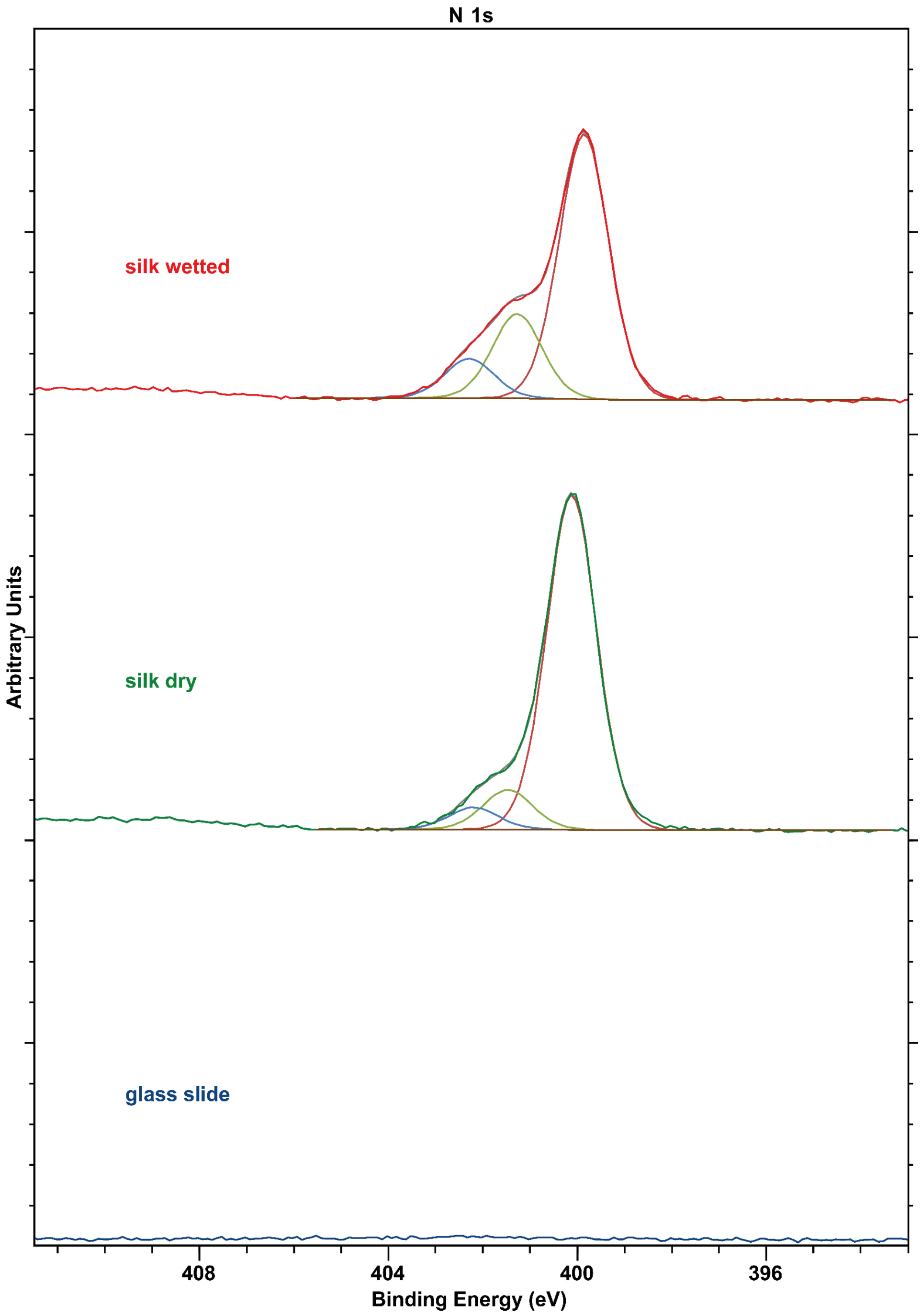

Figure S19. *XPS high-resolution N 1s spectra of high CA silk films (wetted and dry) and a glass slide.*

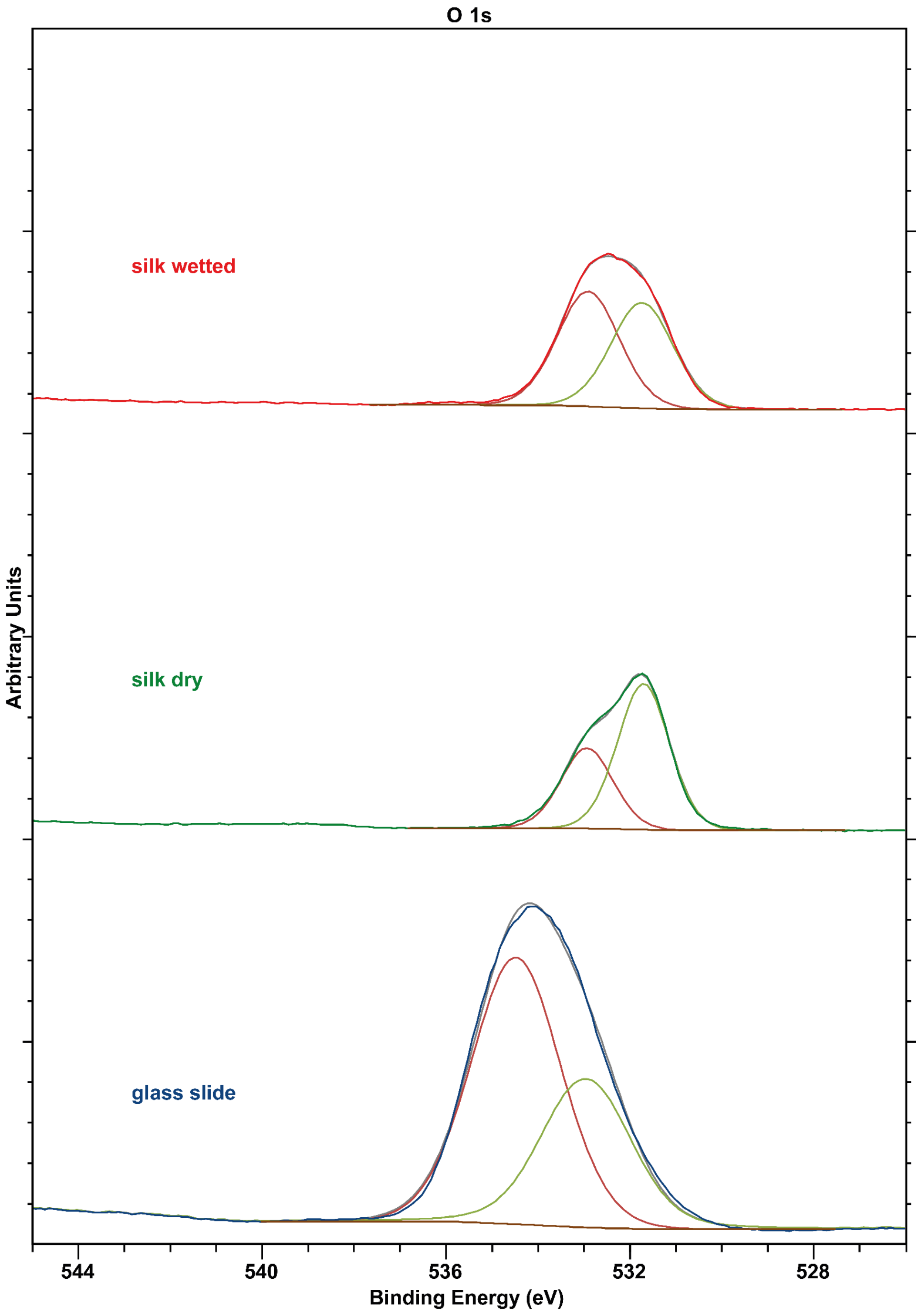

Figure S20. *XPS high-resolution O 1s spectra of high CA silk films (wetted and dry) and a glass slide.*

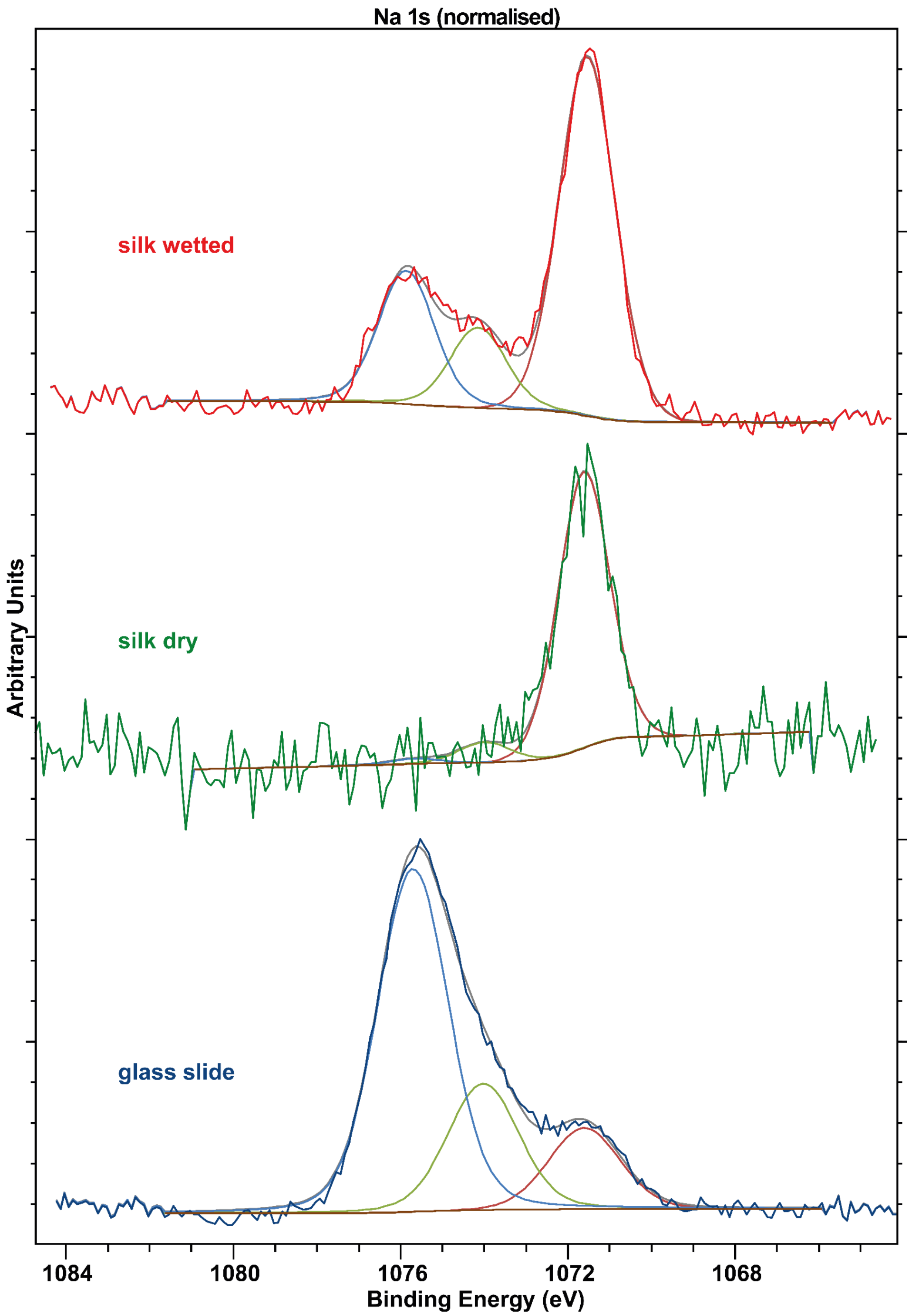

Figure S21. *XPS high-resolution normalised Na 1s spectra of high CA silk films (wetted and dry) and a glass slide.*

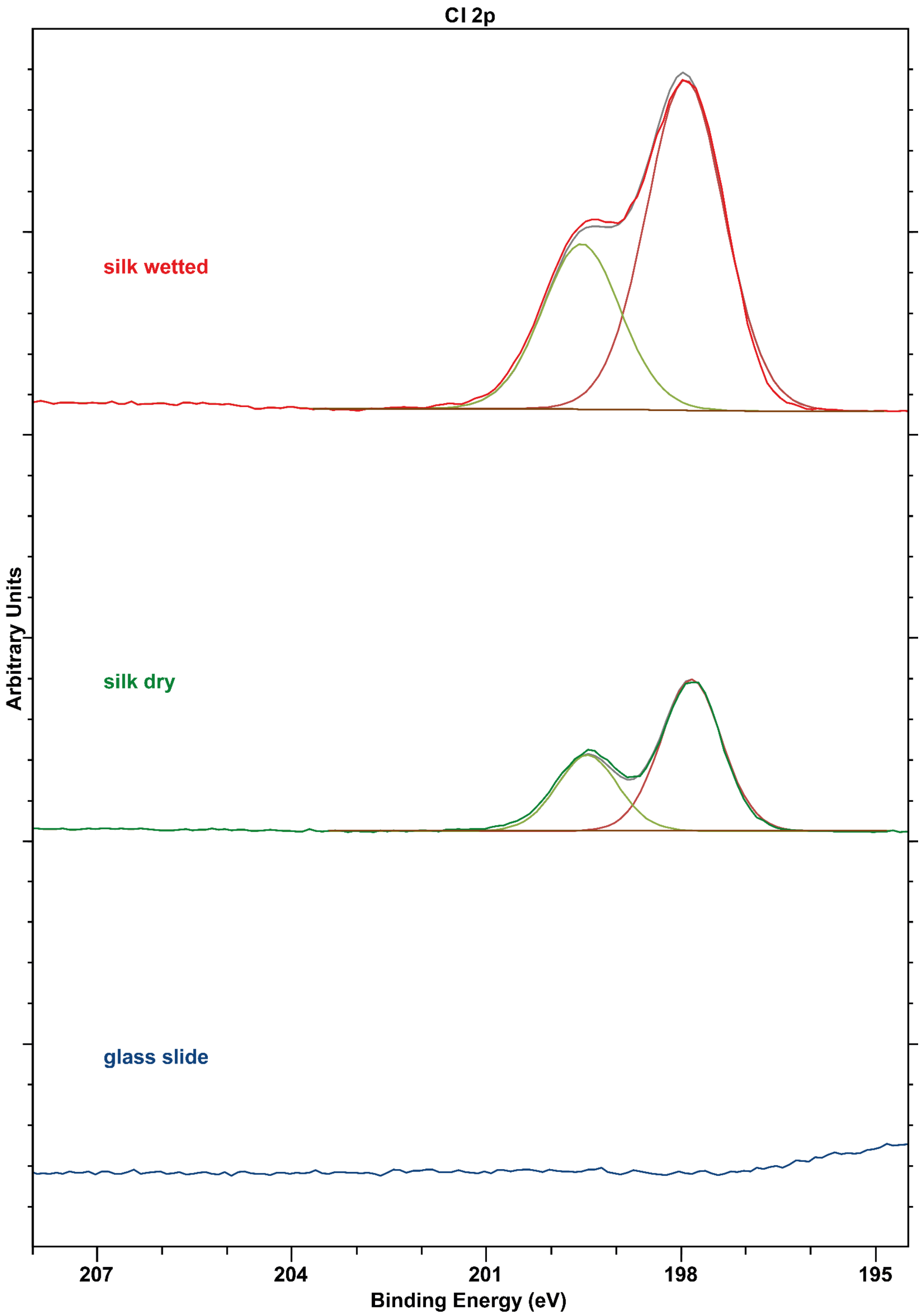

Figure S22. *XPS high-resolution Cl 2p spectra of high CA silk films (wetted and dry) and a glass slide.*

Effect of methanol treatment on the contact angle of the silk film

Post-treatment with methanol was attempted to reduce the disrupting effect of water on the silk films. Silk films were prepared from 0.3 mg/ml of Crys-ADF3-Crys in 0.5 mM Tris-HCl, and incubated at 80% RH, as described in the Materials and methods section. Some of the silk films were then treated with 100% methanol and dried at RT. The CAs of the non-treated and methanol treated silk films were measured (**Figure S23**). The methanol treatment decreased the CA of the silk film from an average of 123° (without treatment) down to 87°, likely due to disrupting hydrophobic structures on the surface of the silk film.

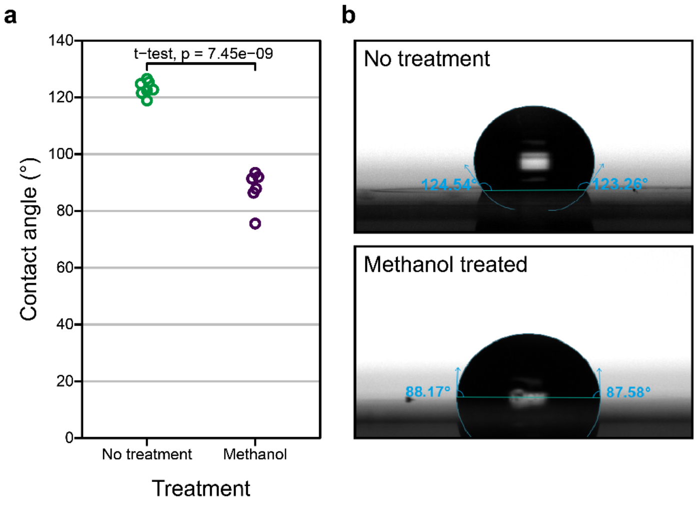

Figure S23. *Effect of methanol post-treatment on the contact angle of the silk film. (a) Contact angle measurement without and with the methanol treatment. (b) Example images of the corresponding contact angle measurement.*
